# Inter-array cabling optimization in offshore wind power plants, a new reliability indicator for radial grids

**DOI:** 10.12688/openreseurope.16716.1

**Published:** 2023-12-01

**Authors:** Ramon Abritta, Alexey Pavlov, Damiano Varagnolo, Børre Tore Børresen

**Affiliations:** 1 Department of Geoscience and Petroleum, Norwegian University of Science and Technology, Trondheim, Trøndelag, 7031, Norway; 2 Department of Engineering Cybernetics, Norwegian University of Science and Technology, Trondheim, Trøndelag, 7034, Norway; 3 Research Center, Equinor ASA, Trondheim, Trøndelag, 7053, Norway

**Keywords:** cabling optimization, radial grids, offshore wind power plants, reliability, renewable energy

## Abstract

This paper presents a radial collection grid optimization approach to support investment decisions into offshore wind power plants (OWPPs). The proposed methodology opportunely combines different optimization criteria and enables trading off cost with power capacity risk (PCR), which is defined as a cumulative power flow summation that reflects the consequences of cable failures. The method strives for a straightforward formulation and solving approach. We define the optimization problem as a mixed-integer linear programming formulation. To construct a relevant case-study, we consider an OWPP from the literature, and validate results via quasirandom Monte Carlo simulations. The numerical results reveal a strong correlation between the proposed PCR and the *expected energy not supplied* reliability index.

## Plain language summary

As part of the planning of an offshore wind power plant, experts need to decide not only where to place the wind turbines, but also how exactly to interconnect them and bring power to the offshore substation. In other words, "Which cable should go from which turbine to which turbine? Should it go directly to the substation instead?". These decisions impact the development planning in different ways such as cost with equipment, cost with installation, and the level of reliability of the power plant in terms of the generated energy. This paper analyzes the trade-offs between cost and reliability since increasing one usually implies decreasing the other. In addition, this work proposes a new metric that seeks to simplify the way to address reliability when optimizing the interconnection cables, which is an important contribution given how complex this task generally is. For the reported case-study, the obtained results indicate that the proposed metric is capable of effectively addressing reliability despite its mathematical simplicity.

## 1 Introduction

Over the last decades, the rising global average temperatures ^1^ have been driving efforts to break the warming trend and secure a safer environment for future generations. The European Commission has set the goal of reducing greenhouse gas emissions by at least 55% by 2030. In addition, Europe intends to become climate neutral by 2050 ^2^. In the energy context, significant investment has been made on renewable energy sources (RES) ^3^ as replacements to hydrocarbon-based generators. Intermittent RES, such as wind and solar, have been the main target of investments due to their availability and clean process of power generation ^4^. The global offshore wind capacity more than tripled from 2017 to 2022, reaching 64.3 GW by the end of 2022 ^5^.

Although most of the OWPPs costs come from the wind turbines (WTs) ^6^, a significant portion relates to the collection grid (CG), i.e., the cables that link the WTs to the offshore substation (SS). For instance, Nieradzinska *et al*. ^7^ reported a CG cost of 12.9% regarding the total costs of Dogger Bank. Pérez-Rúa *et al*. ^8^ claim that cables are responsible for 11%, or more, of the total levelized cost of energy.

Cabling optimization, also known as collection grid optimization, usually regards reliability and costs ^9^. The former can be quantified by different metrics. However, it generally relates to the expected capability of the OWPP to generate a certain share of the available energy potential. The latter can refer to costs from purchase, installation, protection, maintenance, power losses, or a combination of these.

The *expected energy not supplied* ( *EENS*) is an index commonly utilized to assess the reliability of electrical power systems ^10– 12^. As the name describes, this metric quantifies how much energy one can expect that the OWPP will not supply, which is due to events where equipment were not available. Regarding costs, they will mostly depend on the lengths and types of the cables to be installed. A simple approach to quantify the length of a cable linking two components is to utilize the Euclidean distance. However, real lengths can greatly depend on the seabed bathymetry ^9^. Ahead we present a brief review of CG optimization papers.

Wu *et al*. ^13^ utilized the Charged System Search algorithm to optimize the layout of an OWPP. A mixed-integer linear programming (MILP) approach minimized the purchase cost of the radial CG. The authors evaluated reliability post-optimization. Gong, Kuenzel and Pal ^14^ optimized a CG considering a three-dimensional installation surface, and investigated string, ring and multiloop structures. A Particle Swarm algorithm determined the SS location. The authors claimed that the proposed multiloop setup is cost-optimal when failure rate and mean time to repair (MTTR) are relatively high. Pérez-Rúa *et al*. ^8^ studied ring and radial CGs and addressed reliability via stochastic programming. Different scenarios were defined according to cables failures probabilities. The authors stated that ring grids tend to be more propitious for large projects, whereas small OWPPs benefit more from radial grids. Pérez-Rúa *et al*. ^15^ presented an heuristic to reduce the solution search space and globally optimize CGs of large OWPPs. Several case-studies demonstrated the effectiveness and efficiency of the approach. Abeynayake *et al*. ^16^ brought a holistic method to assess the reliability of large OWPPs with radial CGs. Markov models represented the reliability of the equipment. Paul and Rather ^17^ proposed a clustering approach to group WTs, which then formed ring CGs. The authors also studied different forms of connecting multiple OWPPs through HVDC grids. A Monte Carlo algorithm (MCA) estimated *EENS*. Song *et al*. ^18^ proposed a hybrid heuristic to address CG optimization by considering costs from purchase, installation, and power losses. In the presented case-study, the method has outperformed other heuristics.

From the reviewed papers, Wu *et al*. ^13^ did not address reliability as part of the optimization process, whereas 18 did not consider reliability at all. Compared to the remaining studies, this work seeks to reduce the complexity of the solving method regarding the inclusion of reliability aspects.

This paper writes the problem as a MILP formulation, including technical constraints such as the cable-crossing prohibition and the adequacy of power flow. The multi-criteria objective function considers the total cable purchase cost and a proposed power capacity risk (PCR). An quasi-random MCA considering 1000 yearly simulations subjected to wake effects reveals the strong correlation between the proposed PCR and the widely used *EENS* index. We highlight the following contributions from this paper:

- A simple and easily replicable MILP approach to obtain and analyze solutions to radial CGs, both from the perspective of purchase cost and reliability. This is a significant contribution since no study has yet addressed CG reliability in a full MILP approach, as far as the authors know;
- A power risk quantifier that: relies exclusively on affine equations, can be easily inserted as an optimization criterion, overlooks the wind profile, and strongly correlates with *EENS*.

As a remark, the focus of this paper is on the proposed PCR as a relevant reliability indicator for radial grids. Therefore, the paper reduces complexity by neglecting seabed bathymetry and inter-array power losses. For the same reason, the study quantifies costs by considering only purchase expenses.

Section 2 describes the problem by presenting the formulation and introducing the proposed PCR. Section 3 provides the utilized methods for CG optimization and *EENS* estimation. Section 4 presents the obtained results. Section 5 discusses the findings and their potential relation with other studies. Section 6 enunciates our conclusions and intended future research.

## 2 Problem description

This paper utilizes a MILP formulation to carry out the CG optimization considering radial systems with one substation. Since the focus is on the cabling problem, we assume the layout to be established, i.e. the WTs and SS locations have been decided. We address purchase cost and the proposed PCR in a multi-criteria optimization. From the cost perspective, the formulation is based on 13. In a post-optimization stage, we carry out Monte Carlo simulations to assess the reliability of the obtained CGs and analyze the results against the defined PCR. The following subsections describe each of the mentioned parts of the study.

The objective function ( _1_) represents the purchase cost of the cables to be installed. The function depends on the lengths of cables and their prices, as described by Equation 1.


$$F_{1}=\sum_{i\in N_{wt}}\sum_{j\in N_{wt,ss}}\sum_{c\in N_{ct}}L_{ij}\cdot p_{c}\cdot u_{i,j,c},\;(1)$$

where: *N _wt_* is the set of WTs; *N*_*wt*, *ss*_ is the set of WTs and SS; *N _ct_* is the set of possible cable types; *L _ij_* is the length, in kilometer, of the cable connecting *i* to *j*; *p _c_* is the price per kilometer of type *c* cable; and *u*_*i*, *j*, *c*_ is a binary variable that indicates if the type *c* cable linking *i* to *j* is installed ( *u*_*i*, *j*, *c*_ = 1) or not ( *u*_*i*, *j*, *c*_ = 0).

From the *x* and *y* coordinates of WTs and SS, we calculate *L _ij_* as in Equation 2. For simplification, we assume the Euclidean distances to be approximately equal to the real distances.


$$L_{ij}=\sqrt{{\left(x_{i}-x_{j}\right)}^{2}+{\left(y_{i}-y_{j}\right)}^{2}},\forall i\in N_{wt},\forall j\in N_{wt,ss}\;(2)$$

As a sequence, we present constraints describing the cable connectivity of each WT, the cable-crossing prohibition, the power flow, the capacity of cables, and the number of direct connections to the SS. Equation 3 states that an element *i* must connect to a single element *j* with a single type *c* cable.


$$\sum_{j\in N_{wt,ss}}\sum_{c\in N_{ct}}u_{i,j,c}=1,\forall i\in N_{wt},\;i\neq j\;(3)$$

The cable-crossing prohibition defines another CG optimization constraint. Bauer and Lysgaard ^19^ state that cable-crossing may cause excessive heating that would require the cables to have insulation against each other. Furthermore, since one cable would be buried deeper than the other, maintenance would become more costly. To address this constraint, let us consider two different pairs of cables, *ij* and *mn*, with *i*, *m*, *n* ∈ *N _wt_* and *j* ∈ *N*_*wt*, *ss*_. Then, we execute the following procedure ^13^:

- Establish linear relations from *i* to *j* and *m* to *n* with Equation 4 to Equation 7 ( *x* and *y* denote the coordinates).
$$\;a_{ij}=(y_{j}-y_{i})/(x_{j}-x_{i})\;(4)$$
$$\;b_{ij}=y_{i}-a_{ij}\cdot x_{i}\;(5)$$
$$\;a_{mn}=(y_{n}-y_{m})/(x_{n}-x_{m})\;(6)$$
$$\;b_{mn}=y_{m}-a_{mn}\cdot x_{m}\;(7)$$
- Subject *m* and *n* to the *ij* relation, whilst also subjecting *i* and *j* to the *mn* relation, as in Equation 8 to Equation 11.
$$f_{ij,x_{m}}=a_{ij}\cdot x_{m}+b_{ij}\;(8)$$
$$f_{ij,x_{n}}=a_{ij}\cdot x_{n}+b_{ij}\;(9)$$
$$f_{mn,x_{i}}=a_{mn}\cdot x_{i}+b_{mn}\;(10)$$
$$f_{mn,x_{j}}=a_{mn}\cdot x_{j}+b_{mn}\;(11)$$
- Verify if the statement described by Equation 12 is true, meaning that cables *ij* and *mn* would cross if installed, or false, meaning that crossing would not occur.
$$(y_{m}-f_{ij,x_{m}})\cdot (y_{n}-f_{ij,x_{n}})<0\wedge (y_{i}-f_{mn,x_{i}})\cdot (y_{j}-f_{mn,x_{j}})<0\;(12)$$
- If Equation 12 holds, the optimization problem must include one of the constraints in Equation 13 to Equation 14.
$$\sum_{c\in N_{ct}}u_{i,j,c}+\sum_{c\in N_{ct}}u_{m,n,c}+\sum_{c\in N_{ct}}u_{j,i,c}+\sum_{c\in N_{ct}}u_{n,m,c}\leq 1,\;\text{if}\;j\;\text{is}\;\text{not}\;\text{the}\;\text{SS}.\;(13)$$
$$\sum_{c\in N_{ct}}u_{i,j,c}+\sum_{c\in N_{ct}}u_{m,n,c}+\sum_{c\in N_{ct}}u_{n,m,c}\leq 1,\;\text{if}\;j\;\text{is}\;\text{the}\;\text{SS}.\;(14)$$

The presented step-by-step ensures that no cable-crossing occurs in the optimized CG. We highlight that this procedure must be done for all combinations of *i*, *j*, *m*, *n* in which all the elements are different from each other. If two or more indexes represent the same element, no crossing will take place, regardless of the procedure.

Equation 15 calculates the power flow, whereas Equation 16 simultaneously secures that the cable type can tolerate the maximum power and that non-existing cables carry no power.


$$\sum_{j\in N_{wt,ss}}pf_{ij}-\sum_{k\in N_{wt}}pf_{ki}=P_{i}^{nom,pu},\forall i\in N_{wt}\;(15)$$

$$\sum_{c\in N_{ct}}V_{pu}\cdot A_{cap_{c,pu}}\cdot u_{i,j,c}\geq pf_{ij},\forall i\in N_{wt},\forall j\in N_{wt,ss}\;(16)$$

where: *_ij_* and *_ki_* are the power, in p.u., that flow from *i* to *j* and from *k* to *i*; $P_{i}^{nom,pu}$ = 1 is the nominal power of WT *i*, in p.u.; *V _pu_* = 1 is the line voltage of the AC grid, in p.u.; *A*_*cap*_*c*, *pu*__ is the current capacity of type *c* cables, in p.u.

Although not utilized in this paper, Equation 17 limits the number of cables that connect directly to the SS.


$$\sum_{i\in N_{wt}}\sum_{c\in N_{ct}}u_{i,\text{SS},c}\leq M\;(17)$$

where: *M* is the maximum number of cables allowed to directly reach the SS.

In terms of connectivity, *u* is a *n _wt_* x ( *n _wt_* + *n _ss_*) x *n _ct_* matrix, with *n _wt_*, *n _ss_* and *n _ct_* being the number of WTs, substations and cable types, respectively. Equation 18 yields a *n _wt_* x ( *n _wt_* + *n _ss_*) matrix ( *u′*) that does not relate to the cable types. From the interconnection perspective, *u′* defines the CG.


$${u^{\prime }}_{i,j}=\sum_{c\in N_{ct}}u_{i,j,c},\forall i\in N_{wt},\forall j\in N_{wt,ss}\;(18)$$

### 2.1 The proposed power capacity risk

As stated in Section 1, this work seeks to effectively address the reliability of radials CGs in straight-forward and comprehensive way. To model the proposed PCR, Equation 19 defines a new variable ( *δ*) to the problem. Note that *δ _i_* is simply the power that flows through WT *i*’s cable. The new objective _2_ ( Equation 20) is the cumulative sum regarding the power flow of all cables. As will be demonstrated further ahead, this sum reflects the effects of cable failures, and strongly correlates to reliability. In this work, _2_ is the quantification of PCR.


$$\delta_{i}=\sum_{j\in N_{wt,ss}}pf_{ij},\forall i\in N_{wt}\;(19)$$

$$F_{2}=\sum_{i\in N_{wt}}\delta_{i}\;(20)$$

One can interpret that minimizing the cumulative power flow ( _2_) makes the radial system more spread. In other words, more branches are created. Thus, the impact of cable failures decreases due to less power curtailment upon contingencies. By normalizing the purchase cost and PCR, Equation 21, subjected to Equation 3 to Equation 19, defines the new optimization problem.


$$\text{minimize}\;w\cdot {\tilde{F}}_{1}+(1-w)\cdot {\tilde{F}}_{2}\;(21)$$

where: *w* is a weighing factor in [0,1] that balances the two optimization criteria; ${\tilde{F}}_{1}$ and ${\tilde{F}}_{2}$ are the normalized _1_ and _2_ in [0,1]. We explain the normalization procedure in the following section.

## 3 Methods

This section describes the methods utilized to execute the inter-array cabling optimization and to assess the effectiveness of PCR as a reliability indicator. All simulations were done in a 64 GB RAM, Intel ^®^ Core ^TM^ i9-11950H workstation. The following subsections address the two parts of the study.

### 3.1 Inter-array cabling optimization

We retrieved the OWPP with 25 WTs and one SS from 17. The nominal power of the WTs is 7 MW. The nominal line-to-line voltage of the CG is 66 kV. Table 1 lists the cable types and their respective current capacities ^17^ and prices. Paul and Rather ^17^ did not enable the replication of prices, so we estimated them based on 20 (p. 196). As an observation, one needs to divide the current values of Table 1 by the base $\left(7MW/66\sqrt{3}kV\right)$ to obtain *A*_*cap*_*c*, *pu*__ ( Equation 16).

**Table 1.** Cable types, current capacities ( *A _cap _c__*), in Ampere, and prices ( *p _c_*), in MSEK/km.

Cable type ( *c*)	1 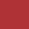	2 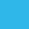	3 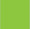	4 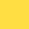	5 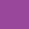	6 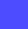	7 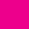	8 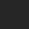	9 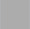	10 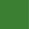
*A _cap _c__*	300	340	375	480	530	590	655	715	775	825
*p _c_*	1.95	2.07	2.19	2.61	2.85	3.18	3.59	4.03	4.53	5.01

The coordinates of the WTs and SS were not disclosed in 17, so we approximated them with the Graph Grabber software. Table 2 shows the extracted data points.

**Table 2.** SS and WTs coordinates, in meters.

	x	y		x	y		x	y
**SS**	2584.44	2015.47	**9**	1167.27	3264.72	**18**	3651.75	2191.55
**1**	67.71	164.19	**10**	977.03	4414.04	**19**	3571.14	3569.30
**2**	156.39	977.99	**11**	2465.14	57.11	**20**	3388.96	4485.43
**3**	29.02	2293.87	**12**	2450.62	1023.20	**21**	4364.37	11.90
**4**	233.78	3364.66	**13**	2507.05	2222.49	**22**	4480.45	1282.57
**5**	140.27	4402.14	**14**	2150.34	3317.39	**23**	4578.80	2115.41
**6**	1268.84	57.11	**15**	2355.50	4466.39	**24**	4488.51	3428.91
**7**	1147.92	1244.50	**16**	3374.45	197.50	**25**	4348.25	4430.70
**8**	1343.01	2522.31	**17**	3485.69	1170.73	-	-	-

We implemented the formulation from Section 2 in the version 1.15.1 of the JuMP environment ^21^ in Julia Programming Language (RRID:SCR_021666), version 1.9.3. We utilized version 1.6.1 of HiGHs’ ^22^ Julia wrapper to solve the problem. HiGHs solves linear programming problems with revised simplex and interior point methods, whereas branch-and-price algorithms handle mixed integer programming problem. An optimality gap of 0.1% was used for the whole study. The implemented code is available in 23.

To normalize _1_ and _2_ and enable Equation 21, first we only minimized the cost ( _1_), so that the PCR ( _2_) would be at its maximum non-dominated value (81 p.u.). Any feasible solution with greater _2_ implies a non-minimal _1_, thus not being worth assessing as such solution would be dominated regarding the Pareto front. For clarification regarding multi-criteria optimization studies, the Pareto front is the set of solutions in which no criterion can be improved without worsening at least one of the other criteria. Second, we exclusively minimized PCR, thus achieving the highest non-dominated cost (1.022 · 10 ^8^ SEK). The exclusive PCR minimization neglects costs, meaning that the code can choose cables with unnecessarily high capacities. Therefore, it is necessary to force cables to be of type one in this case, which is enough to handle the power from one WT.

**Figure 1.**
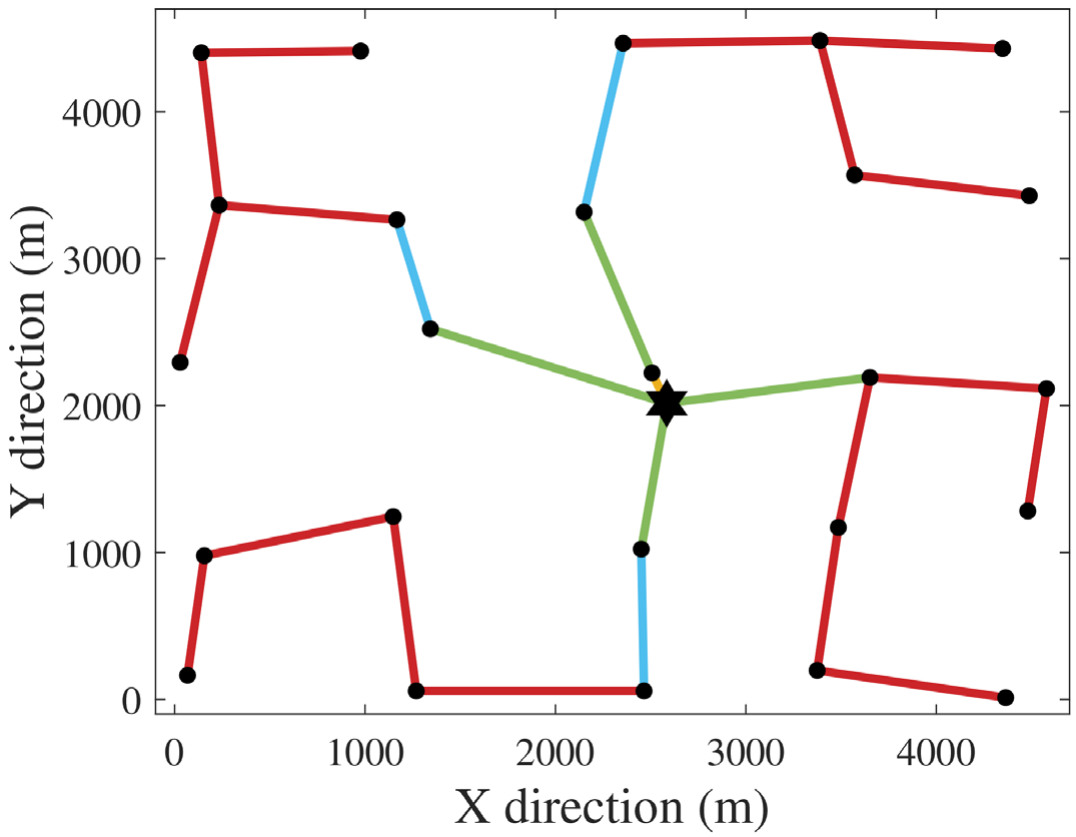
Cost-optimal CG (greatest PCR).

**Figure 2.**
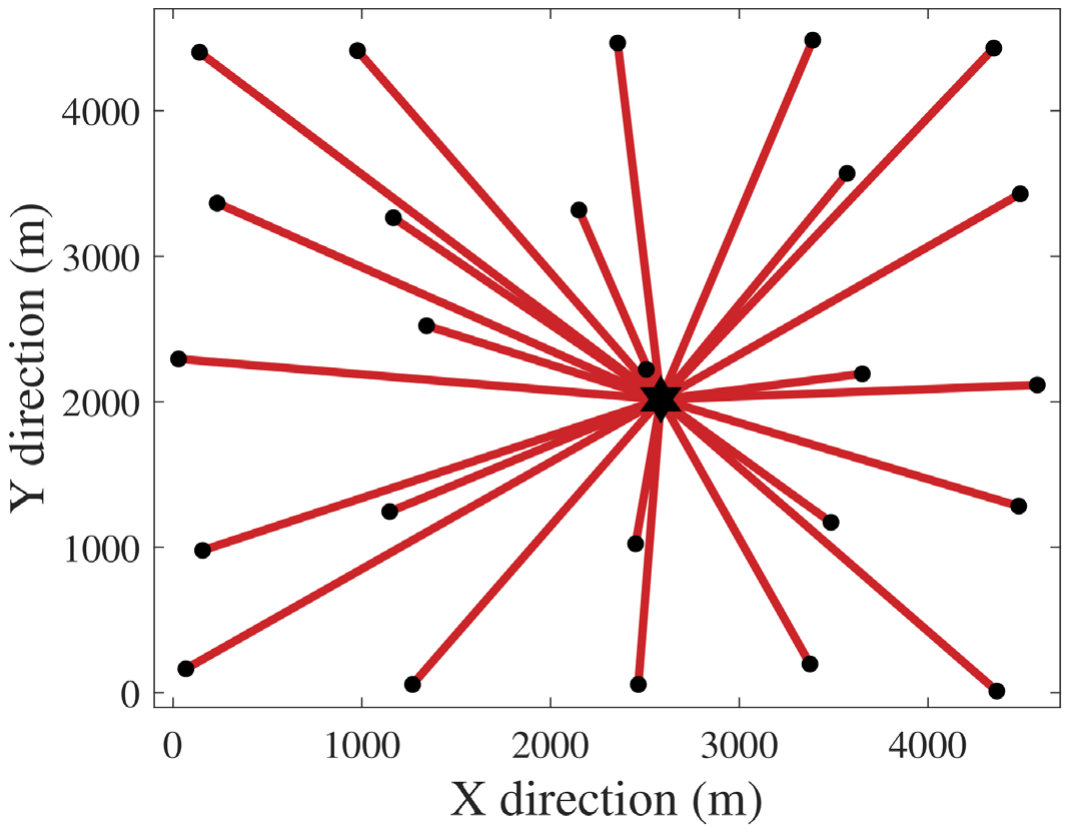
PCR-optimal CG (highest cost).

Figure 1 and Figure 2 depict the CGs for the exclusive cost and PCR optimizations. The hexagram and dots represent the SS and the WTs. The CG of Figure 2 links all WTs directly to the SS since no limitation was set for the number of cables directly reaching the SS ( Equation 17). This CG is technically unfeasible due to protection and connectivity issues that would come with such a topology. However, from a perspective purely based on power risk, a radial CG with one cable per WT would be the safest.

We obtain the normalized objective function ( Equation 21) by making ${\tilde{F}}_{1}$ = _1_/(1.022 · 10 ^8^) and ${\tilde{F}}_{2}$ = _2_/81. To analyze the trade-offs between purchase cost and PCR, we defined 21 cases by reducing the value of *w* ( Equation 21) from 1 to 0 with a step of 0.05. The last case applies *w* = 0.01 instead of *w* = 0 to avoid selecting cables with unnecessarily high capacities.

### 3.2 Quasi-random Monte Carlo algorithm for reliability assessment

The previous section presented the proposed PCR and showed how to pair it with the cable purchase cost formulation to carry out the optimization of radial CGs. To demonstrate the relevance of PCR when addressing reliability, we implemented a quasi-random MCA ^23^ to repeatedly simulate the annual operation of the obtained CGs. We used the average failure rates and MTTR to mimic events of unavailable power.

The term “quasi-random” refers to how to generate the random numbers within the MCA. A quasi-random approach tends to more effectively span the sampling region compared to pseudo-random methods ^24^. Owen ^25^ (Section 15.9) provides an in-depth mathematical example demonstrating the advantages of quasi-random Monte Carlo methods over standard approaches. In essence, a quasi-random procedure tends to better capture the variety of outcomes a variable can have within a bounded region. This work utilizes the QuasiMonteCarlo.jl package, version 1.9.0, to randomize wind directions and WTs or cables failures according to a Sobol sequence.

From 17, the failure rate ( *λ _wt_*) and MTTR ( *r _wt_*) of WTs are equal to 6 occurrences per year and 58.5 hours per occurrence. Regarding the cables, the failure rate ( *λ _c_*) and MTTR ( *r _c_*) are 0.015 occurrences/(km·year) and 1440 hours/occurrence. Over the simulations, we used a discretization length (Δ *t*) of 4.5 hours, i.e., a faulty WT or cable will be out of operation for 13 and 320 Δ *t*’s, respectively. We considered that a year has 1947 Δ *t*’s (365.0625 days), which we will refer to as simulation horizon (SH).

Although the CG optimization only needs the WT nominal power, the MCA validation requires the power curve since different wind speed values imply power outputs that not necessarily will be the nominal power. Since 17 did not disclose the power curve, we fitted polynomials to a down-scaled version of the 8 MW Siemens Gamesa presented and openly available in 26. The cut-in ( *v _ci_*), nominal, and cut-out ( *v _co_*) wind speed values are 3.5, 13, and 25 m/s. Equation 22 describes the resulting power curve function, where *P _g_* and *v* denote the generated power [MW] and wind speed [m/s], respectively.


$$P_{g}=\{\begin{array}{cc} 0, & \text{if}\;v<3.5\\ \alpha_{1}v^{3}+\alpha_{2}v^{2}+\alpha_{3}v+\alpha_{4}, & \text{if}\;3.5\leq v\leq 9\\ \beta_{1}v^{4}+\beta_{2}v^{3}+\beta_{4}v^{2}+\beta_{4}v+\beta_{5}, & \text{if}\;9<v<13\\ 7, & \text{if}\;v\geq 13 \end{array}\;(22)$$

where: *α*_1_ = 5.997 · 10 ^−3^, *α*_2_ = 8.015 · 10 ^−3^, *α*_3_ = *−*0.2556, *α*_4_ = 0.5566, *β*_1_ = 0.02817, *β*_2_ = *−*1.24, *β*_3_ = 20.09, *β*_4_ = *−*140.9, *β*_5_ = 362.8.

**Figure 3.**
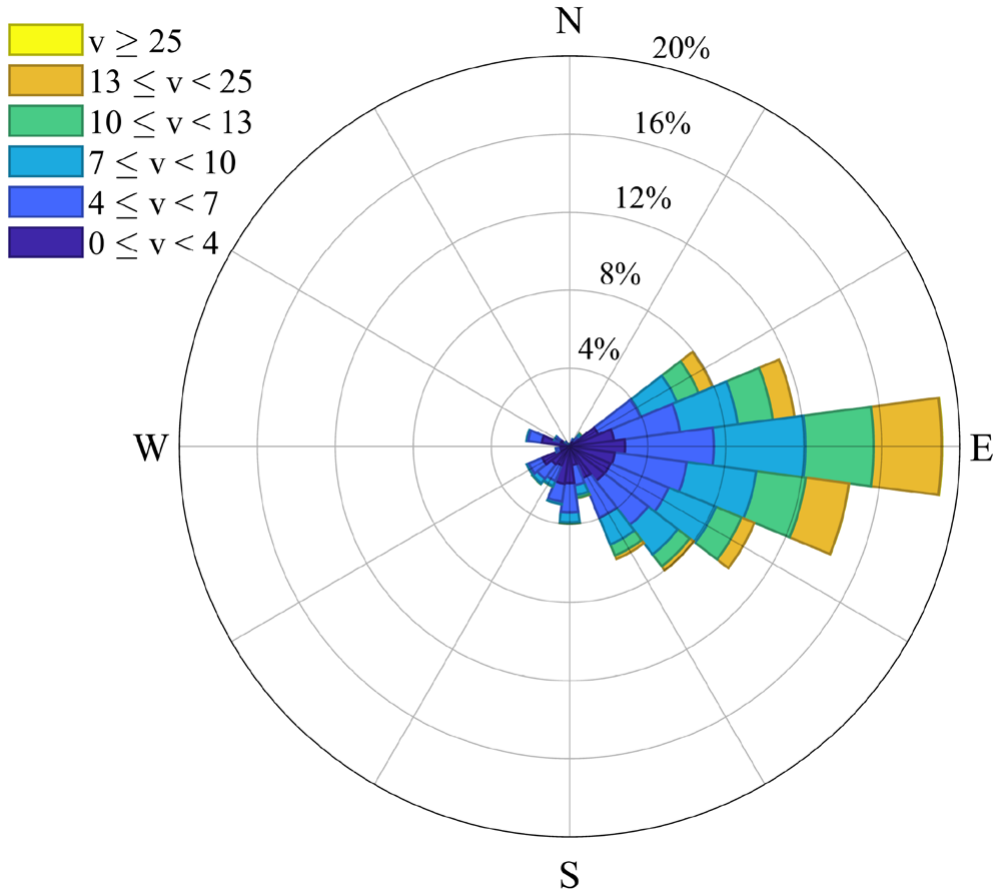
Wind Rose related to Table 3.

Table 3 presents the wind data ^27^. Figure 3 provides a Wind Rose representation created via 28. The wind direction ( *w _dir_*) classes range from lower ( *θ _lb_*) to upper bounds ( *θ _ub_*). Each class has a probability of occurrence ( *ρ*) and two Weibull parameters, *w*_1_ (shape) and *w*_2_ (scale), related to a Weibull distribution function given by Equation 23. We consider that a class 1 direction corresponds to a wind coming from the west.


$$f(v)=\frac{w_{1}}{w_{2}}\cdot {\left(\frac{v}{w_{2}}\right)}^{\left(w_{1}-1\right)}\cdot \exp\left(-{\left(\frac{v}{w_{2}}\right)}^{w_{1}}\right)\;(23)$$

**Table 3.** Wind data ^27^.

Class	*θ _lb_*	*θ _ub_*	*w*_1_	*w*_2_	*ρ*	**Class**	*θ _lb_*	*θ _ub_*	*w*_1_	*w*_2_	*ρ*
**1**	0	15	2	7	0.0003	**13**	180	195	2	10	0.1909
**2**	15	30	2	4	0.0072	**14**	195	210	2	8.5	0.1162
**3**	30	45	2	4	0.0237	**15**	210	225	2	8.5	0.0793
**4**	45	60	2	5	0.0242	**16**	225	240	2	6.5	0.0082
**5**	60	75	2	5	0.0222	**17**	240	255	2	4.6	0.0041
**6**	75	90	2	4	0.0301	**18**	255	270	2	2.6	0.0008
**7**	90	105	2	5	0.0397	**19**	270	285	2	8	0.0010
**8**	105	120	2	6	0.0268	**20**	285	300	2	5	0.0005
**9**	120	135	2	7	0.0626	**21**	300	315	2	6.4	0.0013
**10**	135	150	2	7	0.0801	**22**	315	330	2	5.2	0.0031
**11**	150	165	2	8	0.1025	**23**	330	345	2	4.5	0.0085
**12**	165	180	2	10	0.1445	**24**	345	360	2	3.9	0.0222

When a WT extracts energy from wind, the air flow is affected and the wind speed decreases behind the rotor, hence impacting the power output of WTs that will receive the same air flow. This phenomenon is known as wake effect ^29^. As needed for wake calculations, the rotor diameter is equal to 167 meters. The decay constant ( *d _c_*) and thrust coefficient ( *t _c_*) are site and WT-dependent quantities not trivial to obtain. For simplification, this paper considers values equal to 0.094 and 0.88, respectively ^30^.

We assumed the following regarding failures and unavailability of WTs: a WT failure relates to an event downstream of the transformer that connects the WT to the inter-array grid. Thus, when a WT experiences failure, a circuit breaker isolates it from the grid, and its cable remains available. As a limitation of this paper, failures of transformers, circuit breakers and busbars were not considered. In our analysis, if component A is downstream of B, it means that A is electrically more distant from the SS compared to B. For instance, the SS is upstream of all WTs and cables.

**Figure 4.**
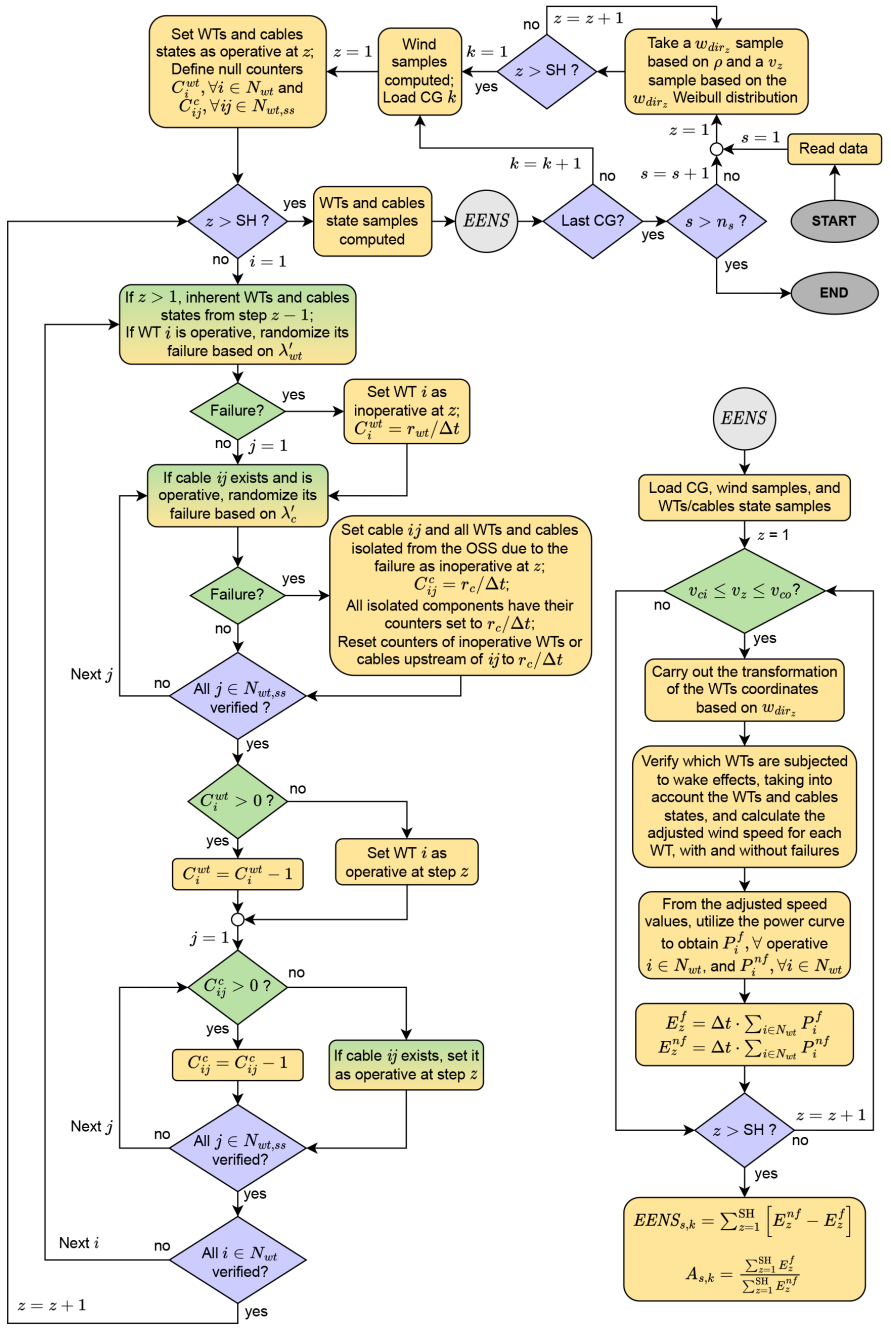
Flowchart of the *EENS* estimation.

Figure 4 illustrates the flowchart of this paper’s MCA. Violet, yellow and green colors indicate, respectively: decisions related to loop structures; direct tasks or calculations; the testing of an “if” statement. Blocks with green to yellow gradient describe actions that involve an “if” statement and others that are direct tasks. The *s*, *z*, *k* indexes denote the current simulation, discretization of the year, and CG under analysis, respectively. The total number of simulations is given by *n _s_*. Ahead we present remarks related to Figure 4.

•  “Read data” refers to WTs and SS coordinates, wind speed model, reliability information (failure rates and MTTR), and parameters of the WT model, in addition to its power curve.

•  A loop takes *w _dir_* [rad] and *v* [m/s] samples to be used over the year discretizations. This process is done separately so that the same wind profile is fed into all CGs to be evaluated.

•  After loading a CG, we create variables related to the states of the WTs and cables and set them as operative (state = 1) in the first discretization. We also define the counter variables, which are responsible for keeping components unavailable according to the associated MTTR. Implementation-wise, a 2D *n _wt_* x SH array represents the WTs states. A 3D *n _wt_* x( *n _wt_* + *n _ss_*) x SH array stores the cables states, with each 2D matrix being a copy of *u′*, though modified according to failures (if a cable *ij* is faulty in discretization *z*, the state matrix in position *ijz* is set to zero).

•  The yearly failure rates *λ _wt_* and *λ _c_* are adjusted to Δ *t*. If a random number in [0,1] is less than the rate, the WT (or cable) is set as inoperative. Its counter variable will store the number of discretizations during which the component will remain unavailable. In a radial grid, a cable failure implies isolation of all downstream components (WTs and cables). Hence, these components are also set as unavailable. Doing so is crucial to avoid randomizing failures of components that are not operative. In this paper, we obtain an undirected graph from the connection matrix *u′*, and then we utilize graph theory to detect isolated components.

•  A cable may fail in a situation where a downstream cable is faulty and unavailable. In such a case, the counter variables of all components that were already isolated must be reset according to the MTTR of the cable currently experiencing failure. Otherwise, the algorithm will restore availability of isolated components.

•  If a WT counter variable is greater than zero in a particular discretization of the MCA, meaning that the component is unavailable, we reduce the counter variable by one, which represents 4.5 hours out of the 58.5 hours MTTR. If the variable is equal to zero, the WT is set as operative. Note that when the counter reaches zero, only in the next discretization the WT is made available again. An analogous procedure is done for the cables.

•  A null *w _dir_* corresponds to a west wind. For different directions, Equation 24 rotates the CG so that the wind comes from west in the transformed system. For instance, if *w _dir_* = π/4 rad, the CG rotates π/4 rad clock-wise.


$$\left[{x^{\prime }}_{j};{y^{\prime }}_{j}\right]=\left[x_{j}\cos(w_{dir})+y_{j}\sin(w_{dir});-x_{j}\sin(w_{dir})+y_{j}\cos(w_{dir})\right],\forall j\in N_{wt,ss}\;(24)$$

•  We used Jensen’s model ^31, 32^ to estimate wake effects. Readers interested in an implementation-oriented formulation of the model are referred to 30. Wake calculations consider the states of the components since inoperative WTs are irrelevant in terms of wake effects. We must also estimate wake neglecting failures to enable the quantification of the unavailable energy.

•  The energy [MWh] at the current discretization considering $\left(E_{z}^{f}\right)$ and neglecting $\left(E_{z}^{nf}\right)$ failures come from the multiplication of the discretization length by the total power output considering $\left(P_{i}^{f}\right)$ and neglecting failures $\left(P_{i}^{nf}\right)$ .

•  After sweeping the whole SH, one can calculate the *EENS* and availability ( *A*) of the current CG for each simulation. Upon execution of all simulations, Equation 25 and Equation 26 provide the mean values ( *EENS* and *A*) for CG *k*.


$${\bar{EENS}}_{k}=\frac{\sum_{s=1}^{n_{s}}EENS_{s,k}}{n_{s}}\;(25)$$

$${\bar{A}}_{k}=\frac{\sum_{s=1}^{n_{s}}A_{s,k}}{n_{s}}\;(26)$$

## 4 Results

**Figure 5.**
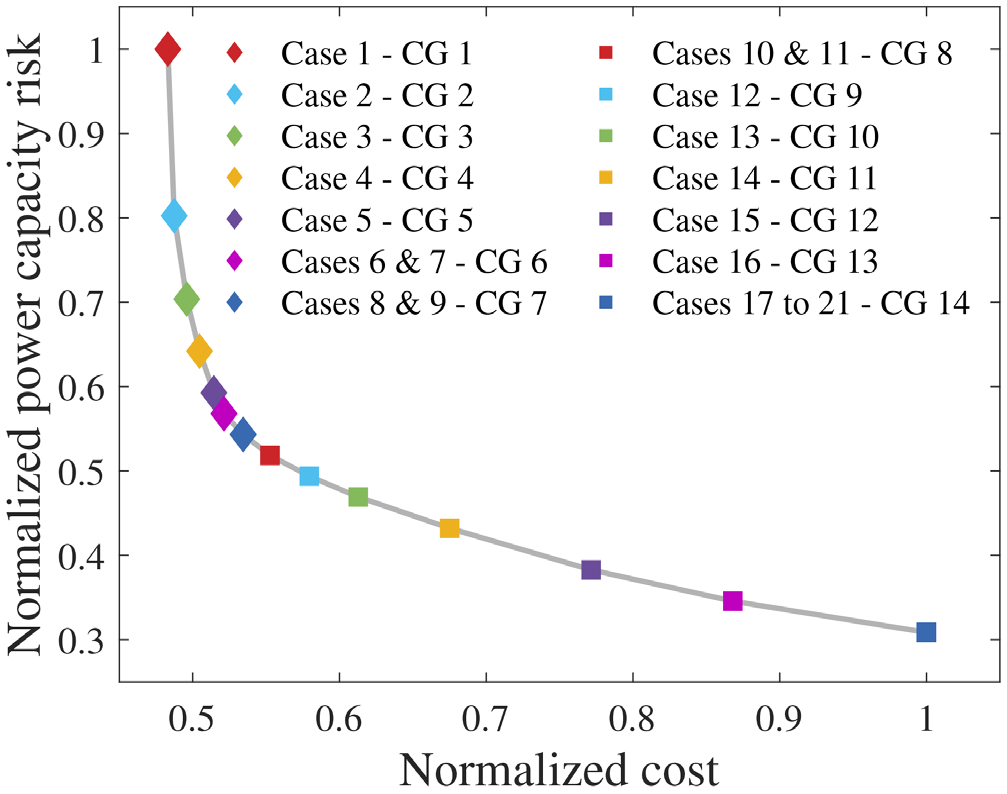
Pareto solutions.

**Figure 6.**
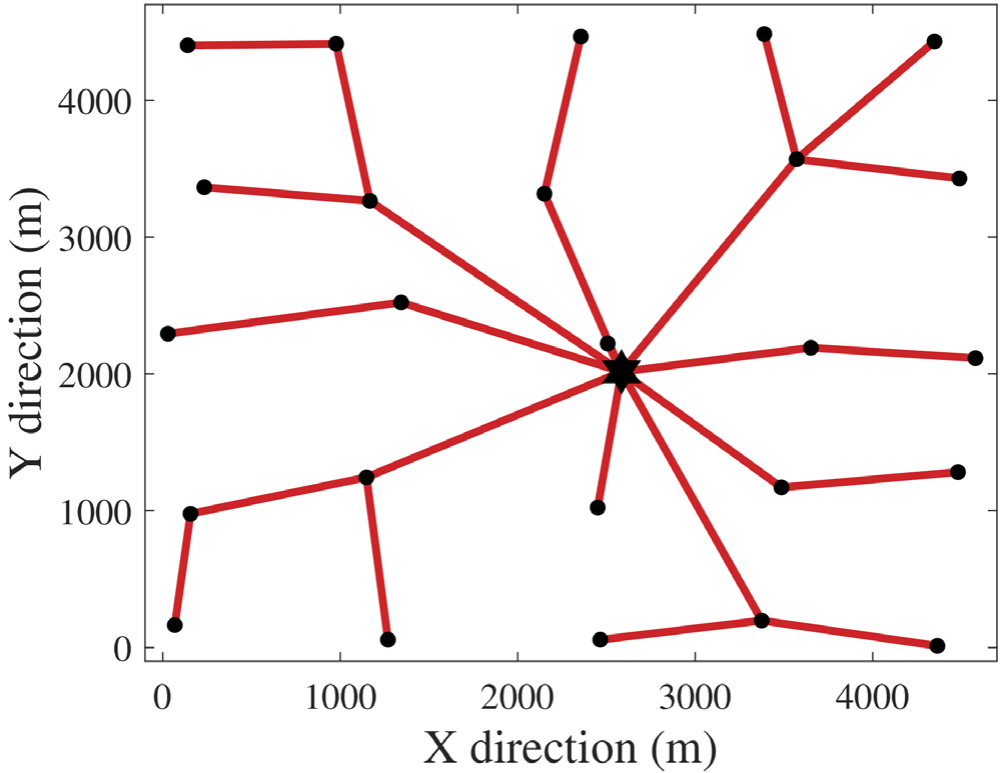
CG 8.

Regarding the solving approach described in subsection 3.1, Figure 5 shows the Pareto solutions obtained by varying the *w* values in Equation 21. As seen, some of the *w* cases yielded the same CG, thus resulting in 14 different CGs. This figure reveals that a significant PCR reduction can be achieved with a small cost increase. For instance, from CG 1 to CG 8, the PCR normalization in [0,1] (nPCR) decreased from 1 to 0.52 (48%), whereas the normalized cost (nC) increased from 0.48 to 0.55 (14.6%). On the other hand, further investment implies significant cost increment, although the PCR reduction does not follow the same proportion. More specifically, from CG 8 to CG 14, nPCR decreased from 0.52 to 0.31 (40.4%), whereas nC raised from 0.55 to 1 (81.8%). Figure 6 illustrates CG 8, which has shown to have an interesting trade-off between the formulated criteria.

Figure 6 demonstrates how minimizing PCR affects the topology of the CG. Compared to the cost-optimal CG 1 ( Figure 1), CG 8 has more branches due to inclusion of PCR in the objective function. In other words, compared to CG 1, CG 8 is less prone to losses of power generation when cable failures occur. Ahead we address the relation between PCR and *EENS*. As a consequence, the potential of the proposed PCR to tackle reliability of radial CGs becomes clear.

**Figure 7.**
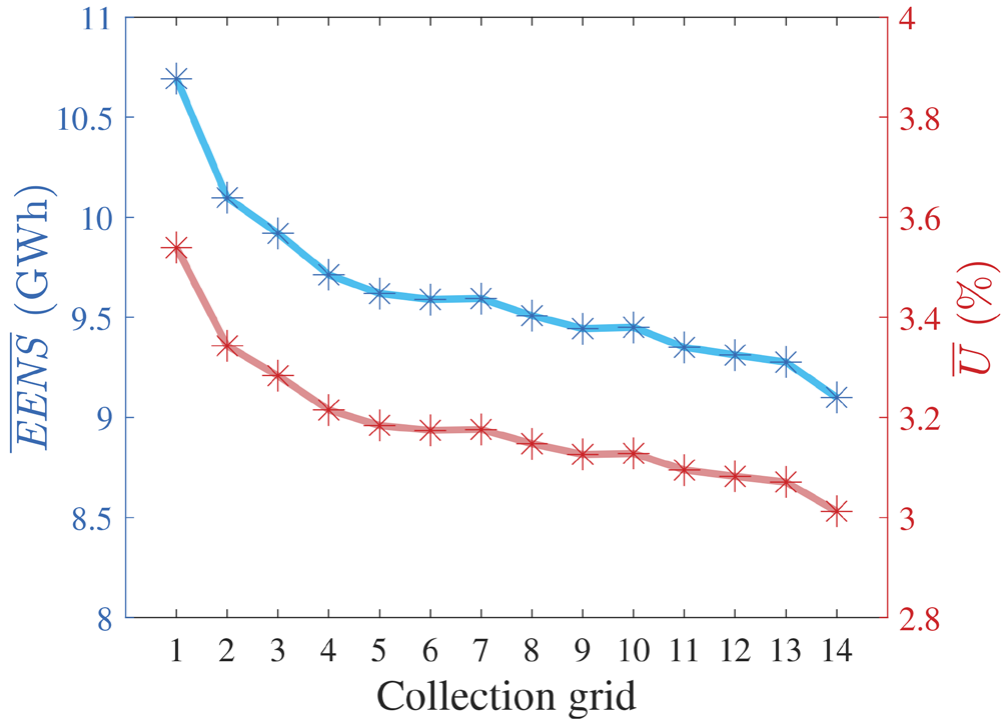
*EENS* and *U* of all radial CGs.

**Figure 8.**
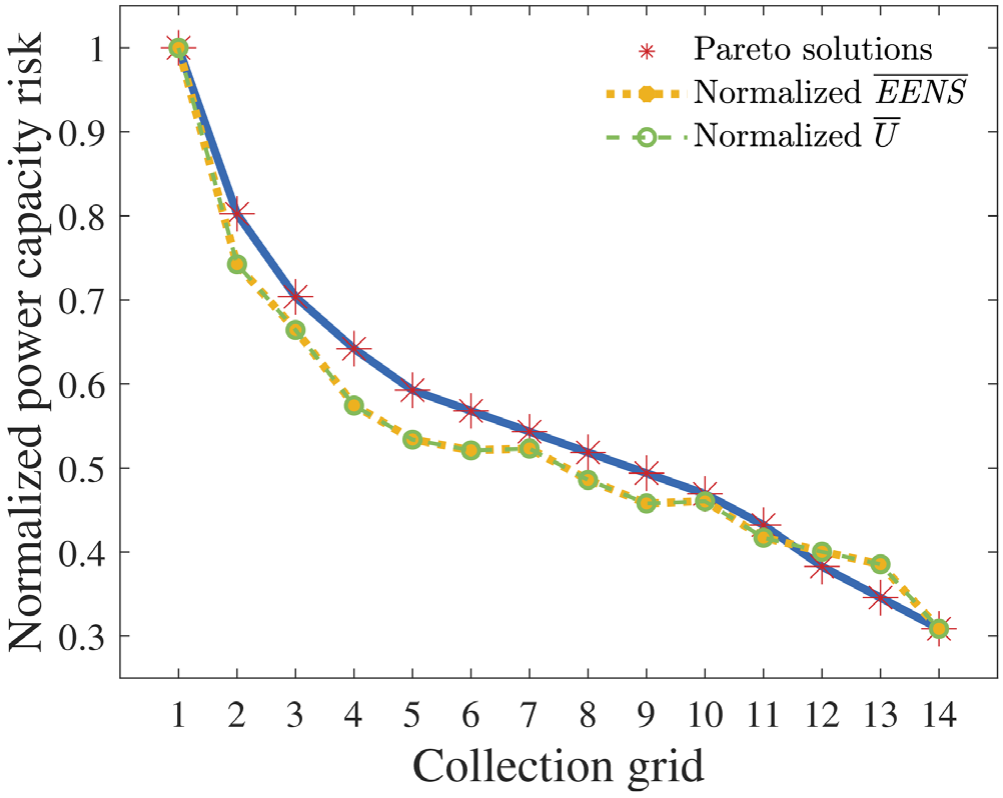
nPCR against the normalized *EENS* and *U*.

We applied the quasi-random MCA from subsection 3.2 to the 14 CGs from Figure 5, with the number of simulations per CG being *n _s_* = 1000. Figure 7 presents the *EENS* and mean unavailability ( *U* = 1 − *A*) curves. Figure 8 normalizes *EENS* and *U* regarding nPCR.

## 5 Discussion

From the previous section, visual inspection of Figure 8 reveals a strong correlation between *EENS* and nPCR. Pearson’s correlation of nPCR with the normalized *EENS* is 0.9706. Therefore, our results suggest that the proposed PCR effectively addressed reliability when optimizing radial CGs under the assumptions made. Some observations are highlighted: (i) an nPCR equal to 1 relates to the *EENS* of the cost-optimal radial CG; (ii) the nPCR calculation does not enable the quantification of the ratio between the *EENS* of a certain CG and the cost-optimal *EENS* ( *EENS _CO_*). For instance, if the nPCR of a non-cost-optimal CG is equal to *σ* ∈ [0, 1], the affirmation that *EENS*/ *EENS _CO_* = *σ* is false. Observation (ii) reveals the weakness of nPCR. Still, the obtained results indicate that addressing PCR strongly correlates with improving the expected reliability of a radial CG.

Zhao *et al*. ^33^ stated that weakening the influence of wind intermittency when assessing reliability is beneficial since the uncertain behavior of wind is difficult to predict. The here proposed PCR has no relation with the wind profile, which is a major strength of the method on top of the strong correlation with *EENS*. Therefore, we argue that PCR can be an important support tool to analyze the trade-offs between cost and reliability of radial CGs.

### 5.1 Brief comparison with a ring CG

**Figure 9.**
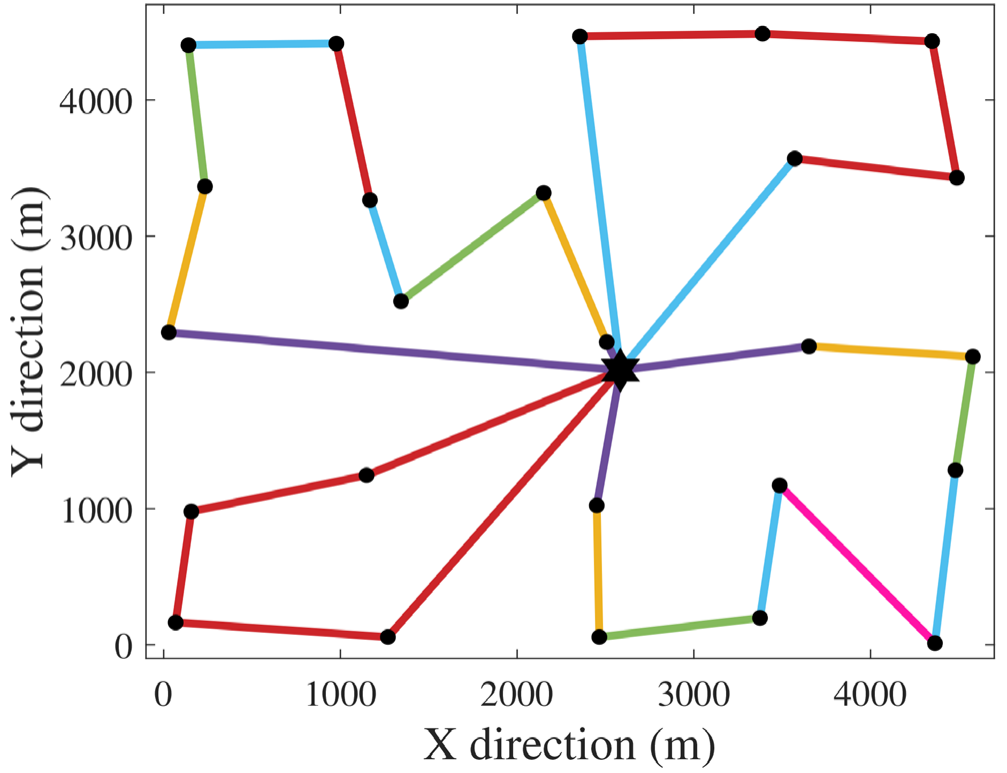
CG 15 ^17^.

**Figure 10.**
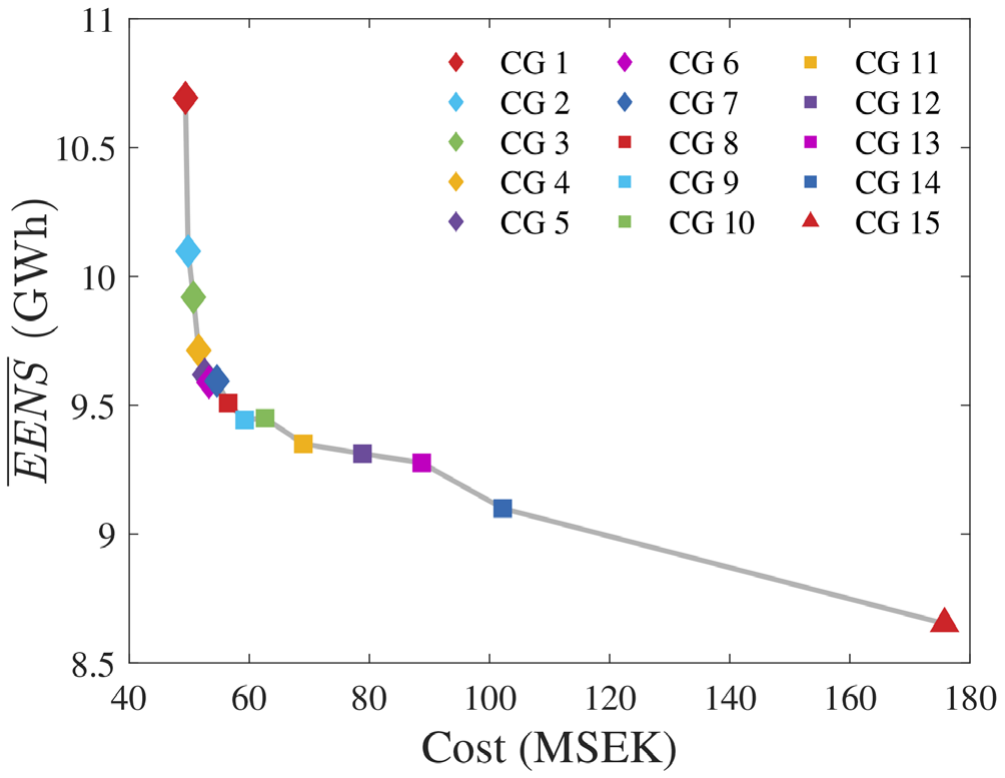
*EENS* versus cost of all CGs.

In 17, which addressed ring CGs, the authors found the CG shown in Figure 9 as the optimal solution to the OWPP under study. This CG will be referred to as CG 15. As stated in Section 3, the cable prices were not provided in 17. However, calculating the purchase cost of CG 15 utilizing the cable prices of Table 2 (which renders a fair comparison) results in a cost of 175.8 MSEK. Ring CGs for the same OWPP will usually be more expensive due to the additional cables required to close the loops. In addition, comparing to Figure 1, we see that the radial CG requires cables of type up to 4, whereas the ring CG uses four type 5 cables and one type 7 cable. To enable a proper comparison, we applied the MCA algorithm to estimate the *EENS* of CG 15. We utilized the same wind samples from the other CGs across 1000 simulations. Figure 10 provides a visual comparison by plotting *EENS* against the cost for all CGs.

Among the radial grids, CG 8 has a good balance between cost and reliability. Comparing it to CG 15 reveals that the total cost increases from 54.6 MSEK to 175.8 MSEK (221% increase), whereas *EENS* decreases from 9.51 GWh to 8.65 MWh (9% decrease). The ring CG requires an additional initial investment of 121.2 MSEK. Given the expected increase of 0.86 GWh in annual energy production (which would lead to additional revenues from energy sales), and the expected lifetime of the OWPP, it becomes relevant to calculate the net present value (NPV) of the project for both alternatives. Such an analysis would provide more managerial awareness regarding the decision of whether it is worth it or not investing into the ring CG. In other words, the estimated NPV would indicate if the increased energy production over the expected OWPP lifetime adds value despite the significant increment to the initial capital investment. As an additional remark, such a study should include other costs, such as the ones from installation, protection, maintenance, and power costs, to enable more realistic results and interpretations.

Since this paper focuses on the proposed PCR and its correlation to the *expected energy not supplied*, addressing the investigation described in the previous paragraph is left for future work.

### 5.2 Considerations on the potential addition of PCR to joint layout and inter-array optimization methods

In the context of OWPPs, *layout optimization* refers to establishing the optimal location (coordinates) of the WTs and SS within a pre-established area. The importance of adequately placing the components comes from the fact that different layouts imply different losses due to wake effects, which should be minimized.

**Figure 11.**
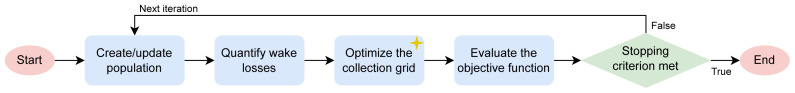
Basic structure of a joint layout and collection grid optimization framework.

From sources with meteorological expertise, it is possible to utilize historical wind measurements to capture the wind speed and direction patterns in a particular site. Given the known wind behavior, experts utilize wake models to simulate the operation of a specific layout, thus estimating the energy that would be lost due to wake effects over a certain period. Metaheuristics are popular methods to address layout optimization problems ^29, 30, 34^. In such approaches, an individual of the population represents a specific layout, which “evolves” over the iterations, up to convergence. The literature presents papers that address layout optimization either as a standalone problem ^34^ or jointly with cabling optimization ^30^. In the latter, prior to the overall evaluation of a specific individual (layout) of the population, the CG is optimized and its cost is incorporated into the objective function. Figure 11 illustrates this approach.

The results from this paper indicate that the proposed PCR might add significant value to the described joint methods. By including the PCR criterion in the CG optimization model, joint methodologies would be capable of addressing reliability aspects of radial grids. However, considerations about potential computational burden issues should be done.

As presented in subsection 2.1, adding the proposed PCR to the mathematical formulation is computationally light given the simple affine equations. However, depending on the number WTs and on the optimality gap, the overall CG optimization, regardless of PCR, can be computationally heavy due to the binary variables. On average, the 21 reported optimization cases ( Figure 5) took 42 seconds to converge. In a metaheuristic approach with 30 individuals subjected to 100 iterations (i.e., 3000 evaluations), approximately 35 hours would be added to the joint layout and inter-array cabling optimization. To a certain extent, this duration is acceptable since the planning problem is not time-sensitive. Nonetheless, computational time could become an issue when addressing OWPPs with a higher number of WTs, which implies additional binary variables.

Computational burden problems could be addressed, or at least alleviated, by: (i) incorporating an heuristic to smartly eliminate part of the binary possibilities (for instance, an optimal CG will hardly connect a WT to another one located at the opposite part of the OWPP area); (ii) relaxing the optimality gap, hence enabling faster MILP convergence at the cost of potentially reducing solution quality; (iii) replacing HiGHs by a commercial solver. Moreover the last item, we used an academic license of Gurobi, version 9.5.1, to solve the 21 optimization cases with the same optimality gap of 0.1%. The mean solving time was 8.7 seconds. Despite the significant improvement, we have chosen to keep HiGHs in 23 to make the code completely open-source.

## 6 Conclusions

This paper presented a novel and simple approach to address the reliability of radial collection grids for OWPP optimization problems formulated as MILP. The cost criterion formulation was based on the literature, and considered technical constraints such as the cable-crossing prohibition. We defined risk according to the proposed PCR, whose formulation relies on affine equations. We explored a case-study considering an OWPP with 25 WTs, one SS, and 10 cable types, and presented the associated Pareto solutions. For validation, we utilized an MCA algorithm to estimate *EENS*. In the context of radial CGs, it has been shown that this widely accepted *EENS* index strongly correlates with PCR, which is the most significant contribution of this paper.

In future research, we intend to: (i) add failure possibilities of other components to the MCA, hence improving the *EENS* estimations and enabling further investigations regarding the correlation with the proposed PCR; (ii) include other cost sources in the formulation, such as installation, protection, maintenance, and power losses; (iii) carry out a comparative study regarding the investment cost and reliability of radial and ring CGs; (iv) assess the viability of the solving approach applied to larger OWPPs, and address the potential computational issues that might arise; (v) seek historical data of real OWPPs as an attempt to validate the proposed PCR against real records of energy not supplied.

## Ethics and consent statement

Ethical approval and consent were not required.

## Data Availability

All data underlying the results are available as part of the article and no additional source data are required.

## Software availability

Archived source code at time of publication: https://www.doi.org/10.5281/zenodo.8413581 ^23^

License: Creative Commons Attribution 4.0 International
