# Inter-array cabling optimization in offshore wind power plants, a new reliability indicator for radial grids

**DOI:** 10.12688/openreseurope.16716.2

**Published:** 2024-05-28

**Authors:** Ramon Abritta, Alexey Pavlov, Damiano Varagnolo, Børre Tore Børresen

**Affiliations:** 1 Department of Geosciences, Norwegian University of Science and Technology, Trondheim, Trøndelag, 7031, Norway; 2 Department of Engineering Cybernetics, Norwegian University of Science and Technology, Trondheim, Trøndelag, 7034, Norway; 3 Research Center, Equinor ASA, Trondheim, Trøndelag, 7053, Norway

**Keywords:** cabling optimization, radial grids, offshore wind power plants, reliability, renewable energy

## Abstract

The inter-array grid relates to a significant share of the investments into an offshore wind power plant (OWPP). Optimizing the cable connections regarding costs and reliability is a mathematically complex task due to the high variety of possible wind and component (wind turbine or cable) failure scenarios. This paper presents a novel mixed integer linear programming approach to support investment decisions into OWPPs by trading off cabling purchase and installation costs with power capacity risk (PCR), which is defined as a length-weighed cumulative power flow summation that reflects the consequences of cable failures. Then, quasi-random Monte Carlo simulations assess the optimized collection grids (CGs) to quantify their levelized cost of energy (LCOE). To construct relevant case studies, this work investigates the real OWPPs Ormonde, Horns Rev 1, Thanet, and London Array, which contain 30, 80, 100, and 175 wind turbines. The results reveal Pearson correlation coefficients around 0.99 between the proposed PCR and the expected energy not supplied. Furthermore, this paper’s findings indicate that minimum-cost CGs do not necessarily present the lowest LCOE.

## Plain language summary

As part of planning an offshore wind power plant, experts need to decide where to place the wind turbines and how exactly to interconnect them and bring power to the offshore substation. In other words, ”Which cable should go from which turbine to which turbine? Should it go directly to the substation instead?”. These decisions impact the development planning in different ways, such as equipment or installation costs and the power plant’s reliability level in terms of the generated energy. This paper analyzes the trade-offs between cost and reliability since increasing one usually implies decreasing the other. In addition, this work proposes a new metric that seeks to simplify how to address reliability when optimizing the interconnection cables, which is an important contribution given how complex this task is. The results of the reported case studies indicate that the proposed metric can effectively address reliability despite its mathematical simplicity.

## Nomenclature

**Table:** 

	Abbreviations
CG	Collection grid
HR1	Horns Rev 1
HVDC	High-voltage direct current
LA	London Array
MCA	Monte Carlo algorithm
MILP	Mixed-integer linear programming
MTTR	Mean time to repair
ORM	Ormonde
OWPP	Offshore wind power plant
PCR	Power capacity risk
TH	Thanet
WT	Wind turbine

**Table:** 

	Parameters and sets
	Set containing the WTs
_0_	Set containing the WTs and substations
	Set containing the cable types
	Set containing the maximum number of WTs that each cable type can support
	Set containing the price per distance of each cable type [M€/km]
*α*	Auxiliary price vector [M€/km]
*nct*	Number of cable types
*L*	Matrix of distances between the elements of _0_ and [km]
*Q _i_*	Maximum number of WTs that can be downstream of element *i* ∈ *N*_0_
*M _c_*	Maximum number of cables directly reaching a substation
	Weight parameter to balance the multi-criteria optimization
*K _h_*	Heuristic parameter, adimensional
*γ _h_*	Heuristic parameter [rad]
*r*_*SS*, *h*_	Heuristic parameter [km]
*σ _h_*	Heuristic parameter [rad]
*θ _l_*	Lower bound of a wind direction class [ *◦*]
*θ _u_*	Upper bound of a wind direction class [ *◦*]
*ρ*	Probability of a wind direction class
*w*_1_, *w*_2_	Shape and scale parameters, respectively, of the Weibull distribution
*v _ci_* , *v _co_*	Cut-in and cut-out wind speed [m/s]
*R _D_*	Rotor diameter [m]
*d _c_*, *t _c_*	Decay constant and thrust coefficient
*λ _wt_*, *λ _c_*	Failure rates of WTs [occurrences/year] and cables [occurrences/year/km]
*L _λ _c__*	Matrix of cable failure rates accounting for the distances [occurrences/year]
∆ *t*	Time step duration in the MCA [hours]
*mttr _wt_*	MTTR of WTs [hours]
*mttr _c_*	MTTR of cables [hours]
*Z*	Set of time steps in an MCA simulation
*S*	Set of Monte Carlo simulations
*d _r_*	Discount rate
*N _y_*	OWPP expected lifetime [years]

**Table:** 

	Variables
*u _*i*, *j*, *t*_*	Binary variable indicating cable *i* ∈ *N*_0_ linking to *j* ∈ *N* and transporting nominal power from *t* WTs
ℱ _1_, ℱ _2_	Cost [M€] and PCR [MWkm] criteria of the objective function, respectively
*w _dir _z,s__*, *v*_*z*, *s*_	Wind direction [rad] and speed [m/s] at the *z*th time-step of simulation *s*
$P_{i}^{f},P_{i}^{nf}$	Power generated by WT *i* considering and neglecting failures, respectively [MW]
$E_{z}^{f},E_{z}^{nf}$	Total generated energy at time step *z* considering and neglecting failures, respectively [MWh]
*EENS*_*s*, *k*_	Expected energy not supplied by CG *k* at simulation *s* [GWh]
*A*_*s*, *k*_, *U*_*s*, *k*_	Availability and unavailability of CG *k* at simulation *s* [%]
*AEP*_*s*, *k*_	Annual energy production of CG *k* at simulation *s* [MWh]
*LCOE _k_*	Levelized cost of energy of CG *k* [€/MWh]
ℐ _*k*_	Total OWPP investment assuming installation of CG *k* [M€]

## 1 Introduction

Over the last decades, the rising global average temperatures ^1^ have been driving efforts to break the warming trend and secure a safer environment for future generations. The European Commission has set the goal of reducing greenhouse gas emissions by at least 55% by 2030. In addition, Europe intends to become climate neutral by 2050 ^2^. In the energy context, significant investment has been made into renewable energy sources ^3^ as replacements for hydrocarbon-based generators. Intermittent renewable sources, such as wind and photovoltaic, have been the main target of investments due to their availability and clean power generation process ^4^. The global offshore wind power capacity grew by 11 GW during 2023, being the second-highest year in history ^5^.

Most of the costs in an OWPP come from the wind turbines (WTs) ^6^. Nonetheless, a significant portion relates to the collection grid (CG), i.e., the cables that connect the WTs to the offshore substation(s). For instance, Nieradzinska *et al*. ^7^ reported a CG cost equivalent to 12.9% of the total Dogger Bank costs. Pérez-Rúa *et al*. ^8^ claim that cable expenses correspond to 11%, or more, of the total levelized cost of energy.

Cabling optimization, also known as collection grid optimization, usually regards reliability and costs ^9^. Different metrics can quantify the former, which relates to the expected capability of the OWPP to generate a certain share of the available energy potential. The latter can refer to costs from purchase, installation, protection, maintenance, power losses, or a combination of these.

The *expected energy not supplied* ( *EENS*) is an index commonly utilized to assess the reliability of electrical power systems ^10– 12^. This metric quantifies how much energy one can expect not to be supplied by the OWPP due to equipment failures.

Cabling costs mostly depend on the lengths and types of the cables and their installation procedure. A simple approach to quantify a cable’s length is to utilize the Euclidean distance. However, real lengths can greatly depend on the seabed bathymetry ^9^. The following paragraph presents a brief review of CG optimization papers.

Cerveira *et al*. ^13^ improved MILP cabling optimization formulations and showcased the approach through OWPPs with 25, 50, and 57 WTs. Shin *et al*. ^14^ optimized the cable layout and substation location through a recursive approach aiming for minimal costs in an OWPP with 80 WTs. Dahmani *et al.* ^15^ utilized a genetic algorithm to assess the cost and reliability of an OWPP comprising 80 WTs. The optimization considered the quantity and location of substations. The authors argue that ring CGs yielded promising results when accounting for lost energy. Wu *et al*. ^16^ utilized the Charged System Search algorithm to optimize the layout of an OWPP with 30 WTs. A mixed-integer linear programming (MILP) approach minimized the purchase cost of the radial CG. The authors evaluated reliability post-optimization. Gong, Kuenzel, and Pal ^17^ optimized a 38 WTs CG considering a three-dimensional installation surface and investigated string, ring, and multiloop structures. A Particle Swarm algorithm determined the substation location. The authors claimed that the proposed multiloop setup is cost-optimal when the failure rate and mean time to repair (MTTR) are relatively high. Pérez-Rúa *et al*. ^18^ presented a heuristic to reduce the solution search space and globally optimize CGs of large OWPPs. Studies with OWPPs ranging from 30 to 108 WTs demonstrated the effectiveness and efficiency of the approach. Zuo *et al.* ^19^ analyzed financial and reliability benefits related to cross-substation utilization in the cabling layout of OWPPs with 96 and 102 WTS. The Fuzzy C-means and Two-Phase Clark and Wright’s saving algorithm performed the CG optimization. The authors claimed that the proposed ring topologies reduced costs with transformers and retained high reliability. Abeynayake *et al*. ^20^ brought a holistic method to assess the reliability of large OWPPs with radial CGs, using Anholt (108 WTs) as the case study. Markov models represented the reliability of the equipment. Zuo *et al*. ^21^ proposed a two-layer method to define WT clusters for ring cabling layouts and optimize the substation location regarding an OWPP with 160 WTs. The approach combines deterministic and metaheuristic steps to achieve minimal costs. Pérez-Rúa *et al*. ^8^ studied ring and radial CGs and addressed reliability via stochastic programming. Different scenarios were defined according to cable failure probabilities. The authors stated that ring grids are usually more propitious for large projects, whereas small OWPPs benefit more from radial grids. Paul and Rather ^22^ proposed a clustering approach to group WTs, forming ring CGs. OWPPs with 20, 25, and 50 WTs were assessed. The authors also studied different forms of connecting multiple OWPPs through HVDC grids. An MCA estimated *EENS*. Song *et al*. ^23^ proposed a hybrid heuristic to address the optimization of a CG with 25 WTs by considering costs from purchase, installation, and power losses. In the presented case study, the method has outperformed other heuristics.

From the reviewed papers, Wu *et al*. ^16^ did not address reliability as part of the optimization process, whereas ^23^ did not consider reliability at all. Compared with the remaining studies, this paper seeks to reduce the complexity of the solving method regarding the inclusion of reliability aspects to the optimization framework.

This work writes the CG optimization problem as a MILP formulation, including technical constraints such as the cable-crossing prohibition and the adequacy of power flow. The multi-criteria objective function considers the total cable purchase plus installation costs and a proposed power capacity risk (PCR). A quasi-random MCA considering 1000 yearly simulations subjected to wake effects assesses the optimized CGs and reveals the strong correlation between the proposed PCR and the widely used *EENS* index. The following contributions are highlighted:

- A simple and easily replicable MILP approach to obtain and analyze solutions to radial CGs, both from the perspectives of costs and reliability.
- A reliability indicator that: relies exclusively on affine equations, can be easily inserted as an optimization criterion, overlooks the wind profile, and strongly correlates with *EENS*.

In the context of MILP formulations, it is possible to stochastically account for equipment failure and wind speed to address CG reliability as part of the optimization. However, representing a large variety of scenarios increases the computational complexity to an extent where solving the problem can become unviable. Thus, such an approach typically requires selecting representative scenarios to reduce the computational complexity in quantifying uncertainties, such as in 8. In this work, a Monte Carlo methodology addresses reliability due to the flexibility and freedom introduced by MCAs. Such algorithms enable the inclusion of probability distributions without the need for scenario selection. In addition, complex phenomena, such as wake effects, can be easily incorporated.

As a remark, this paper focuses on the proposed PCR as a reliability indicator for radial grids assessed via MCA. Therefore, the paper reduces complexity by neglecting seabed bathymetry and inter-array power losses.

Beyond this introduction, Section 2 presents the problem formulation and introduces the proposed PCR. Section 3 describes the utilized methods for CG optimization and *EENS* estimation. Section 4 provides the obtained results and discusses the findings. Section 5 enunciates the conclusions and intended future research.

## 2 Problem description

This paper utilizes a MILP formulation to optimize radial CGs. Since the focus is on the cabling problem, the layout is assumed to be known. In other words. the WTs and substations locations have been established. A multi-criteria optimization addresses costs and the proposed PCR. From the cost perspective, the formulation is based on 13, 18. In a post-optimization stage, Monte Carlo simulations assess the reliability of the obtained CGs and analyze the results against PCR.

### 2.1 Cost-based formulation

**Figure 1.**
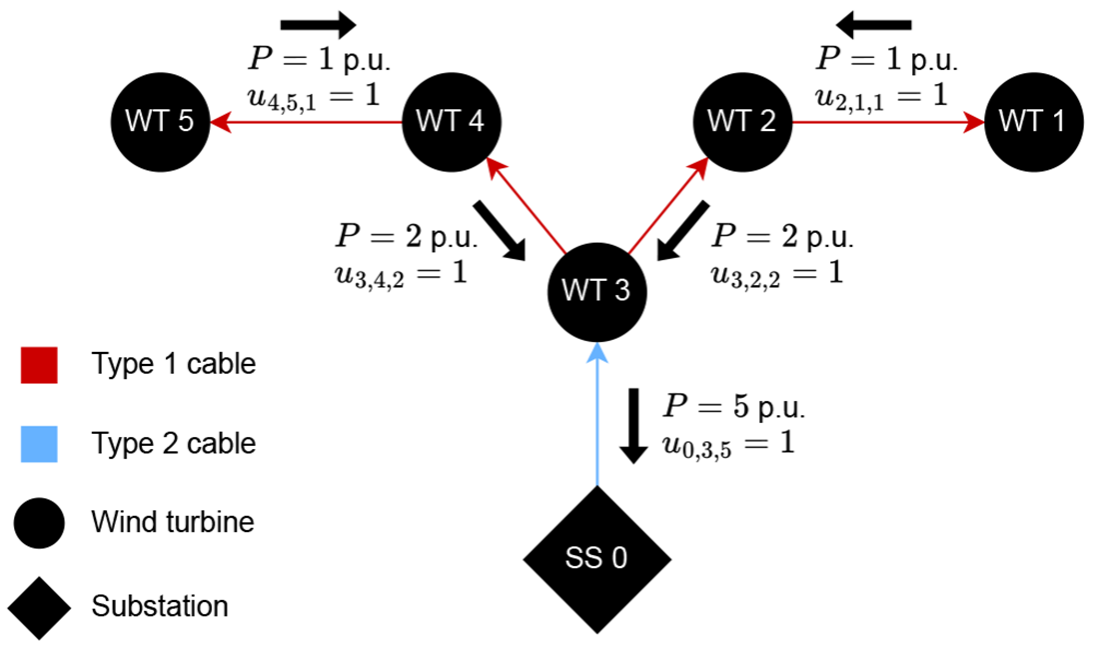
Example of a directed graph representing a CG with 1 substation, 5 WTs, and cables of type 1 and 2 with capacities of up to 3 and 5 p.u., respectively.

Based on 13, 18, the CG is a directed graph spanning from the substation. Binary variables *u*_*i*, *j*, *t*_ represent cables connecting *i* ∈ _0_ to *j* ∈ carrying the nominal power generation from *t* WTs. Note that this convention for the direction of the graph edges implies power flowing from *j* to *i*. The decision variables *u* do not depend on the cable types. If a particular cable transports power from *t* WTs, it is implicit that the optimal cable type is the least costly to support *t* WTs. Figure 1 exemplifies a CG following the described formulation, where binary variables associated with existing cables transporting coherent power are equal to one. Any other binary variable is equal to zero.

Function (ℱ _1_) represents the purchase and installation cost of the cables. The function depends on the lengths of cables and their prices per distance, as described by Equation 1.

$$\;{\text{ℱ}}_{1}=\sum_{i\in N_{0}}\sum_{j\in N}\sum_{t=1}^{Q_{i}}L_{ij}\cdot \alpha_{t}\cdot u_{i,j,t},\;(1)$$

In Equation 1, *α* is an auxiliary cost vector indicating the price per distance of the cables based on the number of downstream WTs. Let us take a set of cable types = { *c*_1_, ... , *c _nct_*} with purchase plus installation prices given by = { *p*_1_, ... , *p _nct_*} and capable of transporting power from up to = { *k*_1_, ... , *k _nct_*} WTs. Observe that set K translates to nominal power transportation capability, in p.u. Vector *α* comes from Algorithm 1.

**Algorithm 1: Pseudo-code linking each nominal power (p.u.) transportation possibility (1, 2, ... , *k _nct_* − 1, *k _nct_*) with the least costly cable type with enough capacity. The values are stored in *α*.**

*aux* = 1

**for** *x* **in** 1 **to** *k _nct_,* **by** 1, **do**

      
**if** *x* ≤ *k _aux_* **then**

            
*α _x_* = *p _aux_*

      
**else**

            
*aux* = *aux* + 1

            
*α _x_* = *p _aux_*

      
**end**

**end**

Still in Equation 1, *Q _i_* is the highest quantity of WTs that can be downstream of *i* ∈ _0_. Note that *Q _i_* = *k _nct_* if and only if *i* is a substation. Otherwise, *Q _i_* = *k _nct_* − 1.

From the *x* and *y* coordinates of WTs and substations, Equation 2 calculates *L _ij_* according to the Euclidean distances among the OWPP components. Matrix *L* contains the lengths of the potentially installed cables.

$$L_{ij}=\sqrt{{\left(x_{i}-x_{j}\right)}^{2}+{\left(y_{i}-y_{j}\right)}^{2}},\forall i\in N_{0},\forall j\in N\;(2)$$

Ahead are the constraints describing the cable connectivity of each WT, the cable-crossing prohibition, the power flow, and the number of direct connections to the sub-stations. Equation 3 states that each *j* ∈ receives one and only one connection, which comes from *i* ∈ _0_ and transports power equivalent to the nominal generation of *t* downstream WTs.

$$\;\sum_{i\in N_{0}}\sum_{t=1}^{Q_{i}}u_{i,j,t}=1,\forall j\in N,i\neq j\;(3)$$

Regarding the cable-crossing prohibition, Bauer and Lysgaard ^24^ state that crossing cables may cause excessive heating that would require the cables to have insulation against each other. Furthermore, since one cable would be buried deeper than the other, maintenance would become more costly. Let us take two distinct cables *ij _1_* and *j _2_j _3_*, with *i* ∈ _0_ and *j*_1_, *j*_2_, *j*_3_ ∈ . If installation implies crossing the cables, either Equation 4 or Equation 5 apply. If *i* is not a substation, the former must be implemented. Otherwise, the latter is valid. It is noteworthy that if any of the elements *i*, *j*_1_, *j*_2_, *j*_3_ is the same as one of the others, it is certain that crossing does not occur.

$$\;\sum_{t=1}^{k_{nct}-1}\left(u_{i,j_{1},t}+u_{j_{2},j_{3},t}+u_{j_{1},i,t}+u_{j_{3},j_{2},t}\right)\leq 1\;(4)$$

$$\;\sum_{t=1}^{k_{nct}}u_{i,j_{1},t}+\sum_{t=1}^{k_{nct}-1}\left(u_{j_{2},j_{3},t}+u_{j_{3},j_{2},t}\right)\leq 1\;(5)$$

Equation 6 ensures that the quantities of WTs upstream and downstream of each WT *j* ∈ are coherent. Since the number of connected WTs is equivalent to the nominal power in p.u., it is critical to observe that Equation 6 also ensures power flow conservation.

$$\sum_{i\in N_{0}}\sum_{t=1}^{Q_{i}}t\cdot u_{i,j,t}-\sum_{q\in N}\sum_{t=1}^{k_{nct}-1}t\cdot u_{j,q,t}=1,\forall j\in N\;(6)$$

Equation (7) imposes a bound on the maximum quantity of cables that can directly reach a substation.

$$\;\sum_{j\in N}\sum_{t=1}^{k_{nct}}u_{i,j,t}\leq M_{c},\forall i\in N_{0}-N\;(7)$$

Equation (8) states that the generation from all WTs must reach the substations. Observe that the *| |* operator denotes the set cardinality.

$$\;\sum_{i\in N_{0}-N}\sum_{j\in N}\sum_{t=1}^{k_{nct}}t\cdot u_{i,j,t}\text{=}\left|N\right|\;(8)$$

If *u*_*i*, *j*, *τ*_ = 1, the number of connections outgoing *j* with *t* downstream WTs must be smaller than or equal to ⌊ ( *τ −* 1) / *t* ⌋, with ⌊ ⌋ denoting the *floor* operation. Based on this property, Cerveira *et al*. ^13^ proposed the constraints in Equation 9 and Equation 10 to reduce the solution space without compromising optimality.

$$\;\sum_{i\in N_{0}}\sum_{\text{τ}=t+1}^{Q_{i}}\left\lfloor \frac{\text{τ}-1}{t}\right\rfloor \cdot u_{i,j,\text{τ}}\geq \sum_{q\in N}\sum_{\text{τ}=t}^{k_{nct}-1}u_{j,q,\text{τ}},\;(9)$$

$$\forall j\in N,\forall t\in 2,\ldots ,k_{nct}-2\;$$

$$\;\sum_{i\in N_{0}-N}u_{i,j,k_{nct}}\geq \sum_{q\in N}u_{j,q,k_{nct}-1},\forall j\in N\;(10)$$

In terms of connectivity, Equation 11 yields a | _0_| x || matrix ( *u′*) that does not relate to the number of downstream WTs. From the interconnection perspective, *u′* defines the CG since it indicates which cables exist. Note that Equation 11 is not a constraint to the optimization problem. However, *u′* is necessary for the upcoming MCA assessments.

$$\;u_{i,j}^{\prime }=\sum_{c=1}^{Q_{i}}u_{i,j,c},\forall i\in N_{0},\forall j\in N\;(11)$$

### 2.2 The proposed power capacity risk

This work seeks to comprehensively address the reliability of radials CGs. To model the proposed PCR, Equation 12 defines a new variable ( *δ*) to the problem. Note that *δ _j_* is simply the nominal power flowing through WT *j*’s cable weighed by the cable’s length. Thus, function ℱ _2_ ( Equation 13) is the cumulative sum regarding the power flow of all cables weighed by their lengths. As demonstrated further, ℱ _2_ reflects the effects of cable failures and strongly correlates to reliability. In summary, ℱ _2_ quantifies PCR.

$$\;\delta_{j}=\sum_{i\in N_{0}}\sum_{t=1}^{Q_{j}}t\cdot u_{i,j,t}\cdot L_{ij},\forall j\in N\;(12)$$

$$\;{\text{ℱ}}_{2}=\sum_{j\in N}\delta_{j}\;(13)$$

The reason for weighing the flowing power according to the cable lengths is that longer cables are more prone to failures. After all, it is usual in the literature to model failure rates of cables as a certain number of occurrences per period per length, such as in 15, 22. Thus, this approach penalizes the utilization of longer cables as these tend to experience failures more often than shorter cables. Subsequent sections show that minimizing the length-weighed cumulative power flow (ℱ _2_) makes the radial system more spread. In other words, more branches are created. Thus, the impact of cable failures tends to decrease due to less power curtailment upon contingencies. By normalizing the cost and PCR, Equation 14, subjected to Equation 3 to Equation 10 and Equation 12, defines the optimization problem.

$$\;\text{minimize}\;\text{W}\cdot {\tilde{ℱ}}_{1}+\left(1-\text{W}\right)\cdot {\tilde{ℱ}}_{2}\;(14)$$

where: is a weighing factor in [0,1] that balances the two optimization criteria; ${\tilde{ℱ}}_{1}$ and ${\tilde{ℱ}}_{2}$ are the normalized ℱ _1_ and ℱ _2_ in [0,1]. The following section explains the normalization procedure.

## 3 Methods

This section describes the methods for inter-array cabling optimization and reliability assessment. The following subsections address the two parts of the study.

### 3.1 Inter-array cabling optimization

This paper utilizes the layouts of four real OWPPs as use cases: Ormonde, Horns Rev 1, Thanet, and London Array. The positions of WTs and substations in the OWPPs are based on 18, 25– 27, and 28, 29, respectively. The coordinates were extracted using the Graph Grabber software and are available in 30. The nominal power ( *P _n_*) of the WTs in the OWPPs are 5, 2, 3, and 3.6 MW, respectively. The nominal line-to-line voltage ( *V _n_*) of the CGs is 33 kV. Based on the current ratings ( *I _n_*) of the cables available in an OWPP, one can obtain set with Equation 15. Pérez-Rúa *et al*. ^18^ performed this procedure for the four OWPPs under study. Table 1 lists the cable types, their maximum number of supported WTs, their prices regarding purchase and installation, and the maximum number of connections to the substations.

$$\;k_{i}=\left\lfloor \frac{\sqrt{3}V_{n}I_{n_{i}}}{P_{n}}\right\rfloor ,\forall i\in \left\{1,\ldots ,nct\right\}\;(15)$$

**Table 1.** Cable data ^18^.

			[M€/km]	*M _c_*
**ORM**	{11,12}	{5,10}	{0.41,0.61}	4
**HR1**	{9,10}	{7,12}	{0.44,0.45}	10
**TH**	{4,5}	{7,10}	{0.44,0.62}	10
**LA**	{1,2,3}	{7,10,13}	{0.36,0.58,0.90}	10

Ormonde has 30 WTs, hence yielding a relatively small optimization problem that can be quickly solved to global optimality. In contrast, Horns Rev 1, Thanet, and London Array have 80, 100, and 175 WTs, respectively. Optimizing the cabling topology of large OWPPs through MILP formulations is a demanding task, given the combinatorial explosion of binary variables. Therefore, it is beneficial to utilize heuristics to exploit system information and reduce the solution region with minimal compromise (preferably none) to global optimality ^18^. The next subsection summarizes an effective heuristic to address large OWPPs, reported by the authors of this paper in 31.

***3.1.1 Heuristic for collection grid optimization, summary of previous work.*** The method in 31 applies different strategies to reduce the computational burden when optimizing cabling topologies in large OWPPs. Strategy 1 establishes circles around all WTs, with the radii equaling the distance from each WT to the closest WT multiplied by a user-defined parameter *K _h_*. For every *i* ∈ outside the circle in which *j* ∈ is the center, *u*_*i*, *j*, *t*_ = 0, ∀ *t*. Furthermore, a user-defined angle *γ _h_* establishes sectors in each WT circle according to the number of substations, with the sectors being oriented in the directions that oppose the substations. For every *i* ∈ inside the circle in which *j* ∈ is the center, if *i* is inside all sectors, *u*_*i*, *j*, *t*_ = 0, *∀t*.

Strategy 2 utilizes a user-defined parameter *r _SS,h_*, in kilometers, that implies *u*_*i*, *j*, *t*_ = 0, ∀ *t*, if *L _ij_* > *r _SS,h_*, with *i* ∈ _0_ − , *j* ∈ . In addition, a small angle *σ _h_*, divisor of 2 *π*, splits the substation-centered circles with radii *r _SS,h_* into several sectors. In each sector, if WT *j* is not the closest one to substation *i*, then *u*_*i*, *j*, *t*_ = 0, ∀ *t*.

Strategy 3 prevents WTs located far from the substations from utilizing cables with the highest capacity. If *L _ij_* > *r _SS,h_*, with *i* ∈ _0_ − and *j* ∈ , then the connections *u*_*j*, *q*, *t*_ = 0, ∀ *t* ∈ { *k*_*nct−*1_ + 1, . . . , *k _nct_*}, ∀ *q* ∈ .

**Figure 2.**
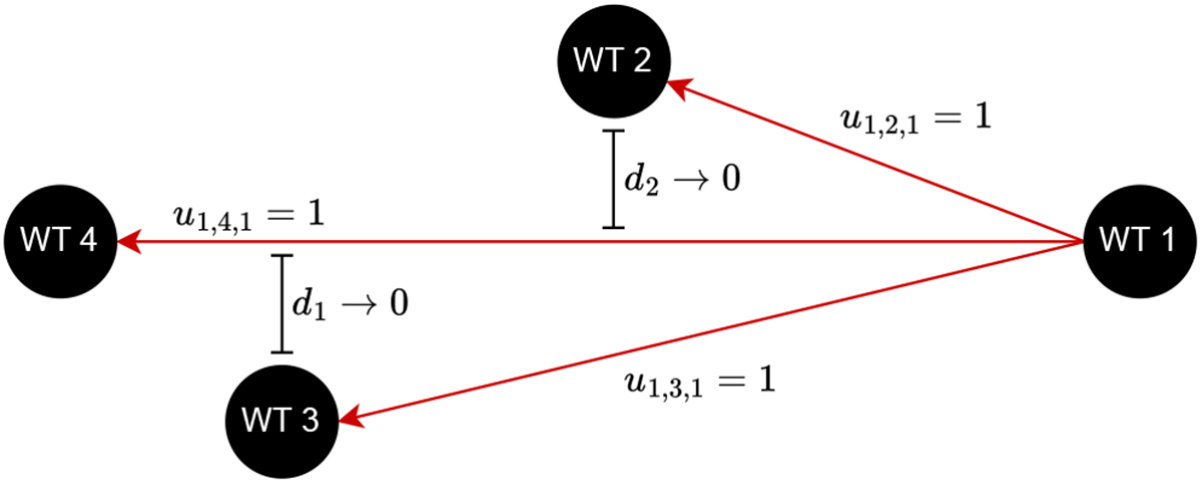
Example of overlapping cable connections not captured by the computations.

Strategy 1 assumes that WTs connect to WTs located in their vicinity and that power transportation opposing the substations’ directions is probably non-optimal. Strategy 2 assumes that substations do not directly link to far-away WTs or two or more WTs that are almost perfectly aligned. Strategy 3 assumes that WTs distant from the substations do not connect to other WTs utilizing the cable with the highest capacity. In this paper, the *σ _h_* part of strategy 2 was extended to also apply to the WT circles. During simulations, it was observed that the calculations often do not capture the alignment of WTs due to distances that tend to zero (orders of magnitude around 10 ^−6^) but that are not exactly zero. The computation “sees” overlapping cables as potential connections, which would not be feasible in reality. Figure 2 exemplifies this situation by showing three connections taken as feasible since *d*_1_ and *d*_2_ are not zero. Utilizing a small *σ _h_* can prevent such occasions.

In this paper, Horns Rev 1, Thanet, and London Array leverage the presented heuristic to achieve solutions in a feasible time. Ormonde utilizes only the *σ _h_*-related parts of the method to eliminate overlapping cables. Ormonde does not suffer from heavy computations, given its small number of WTs. In terms of optimization time, this OWPP can be solved to global optimality in a matter of seconds to a few minutes. The heuristic implementation details are openly available in 31. In addition, the implementation itself is available in 32. This paper merely summarizes the method since it is not the focus of the work.

***3.1.2 Optimization framework.*** The formulation from Section 2 was implemented in the version 1.15.1 of the JuMP environment ^33^ in Julia Language, version 1.9.3. Given the computational complexity of optimizing CGs of large OWPPs, this work uses the commercial solver CPLEX ^34^, version 22.1.1, through an academic license. Version 1.0.1 of CPLEX’s Julia wrapper enables the JuMP-CPLEX interactions. CPLEX addresses linear programming problems with optimizers based on simplex algorithms, whereas branch-and-bound algorithms address mixed integer programming problems.

The whole study used an optimality gap of 0%. Notably, this null gap does not imply global optimality since it regards the reduced problem after applying the heuristic from 31. Table 2 provides the utilized heuristic parameters. Note that Strategy 3 has been completely neglected in all OWPPs for a more conservative approach regarding the cable types. Furthermore, as mentioned, Ormonde only uses the heuristic strategies based on *σ _h_*.

**Table 2.** Heuristic parameters for the CG optimizations.

Parameter	ORM	HR1	TH	LA
***K**_h_*	-	2.5	2.5	2.5
***γ*** *_h_* [rad]	-	π/4	π/4	π/4
***r**_SS,h_* [km]	-	3	2	2
***σ*** *_h_* [rad]	π/90	π/90	π/90	π/90

To normalize ℱ _1_ and ℱ _2_ and enable Equation 14, first, only the cost (ℱ _1_) is minimized, so that the PCR (ℱ _2_) is at its maximum non-dominated value. Any feasible solution with greater ℱ _2_ implies a non-minimal ℱ _1_, thus implying a dominated solution regarding the Pareto front. For clarification, the Pareto front is the set of optimization solutions in which no criterion can be improved without worsening at least one of the other criteria. Second, only PCR is minimized for retrieval of the highest non-dominated cost.

Functions ${\tilde{ℱ}}_{1}$ and ${\tilde{ℱ}}_{2}$ come from dividing ℱ _1_ and ℱ _2_ by the maximum non-dominated cost and the maximum non-dominated PCR, respectively. Then, Equation 14 can be minimized with different values to determine the weights for the optimization criteria, which enable the assessment of the trade-offs between costs and PCR.

### 3.2 Monte Carlo-based reliability assessment

The previous section presented the proposed PCR and its combination with the cost formulation to optimize radial CGs. To demonstrate the relevance of PCR when addressing reliability, a quasi-random MCA repeatedly simulates the yearly operation of the obtained CGs. Average failure rates and MTTR mimic events of unavailable power.

The term “quasi-random” refers to how to generate the random numbers within the MCA. A quasi-random approach tends to more effectively span the sampling region compared with pseudo-random methods ^35^. Owen ^36^ (Section 15.9) provides an in-depth mathematical example demonstrating the advantages of quasi-random Monte Carlo methods over standard approaches. In essence, a quasi-random procedure tends to more effectively capture the variety of outcomes of a variable within a bounded region. This work utilizes the QuasiMonteCarlo.jl package, version 0.3.2, to randomize wind directions and WT or cable failures according to a Sobol sequence.

Carroll *et al*. ^37^ extensively studied failure rates and repair times of WTs, accounting for many components, e.g., gearboxes, generators, blades, transformers, and circuit breakers. Based on 37, this paper assumes the failure rate ( *λ _wt_*) and MTTR ( *mttr _wt_*) of WTs as equal to 8.3 occurrences/year and 88 hours/occurrence. Based on 15, the failure rate ( *λ _c_*) and MTTR ( *mttr _c_*) of cables are 0.015 occurrences/km/year and 1440 hours/occurrence. The MCA simulations in utilize a time step length (∆ *t*) of 8 hours (a common divisor of 88 and 1440). Thus, upon failures, WTs and cables remain out of operation for 11 and 180 time steps, respectively. A year has 1095 time steps, which define set . In this paper, a WT failure relates to an event downstream of the transformer that connects the WT to the inter-array grid. Thus, when a WT experiences failure, a circuit breaker isolates it from the inter-array grid. Therefore, the WT’s cable remains available. If component A is downstream of B, A is electrically more distant from the substation compared with B. For instance, the substation is upstream of all WTs and cables, assuming an OWPP with a single substation.

**Figure 3.**
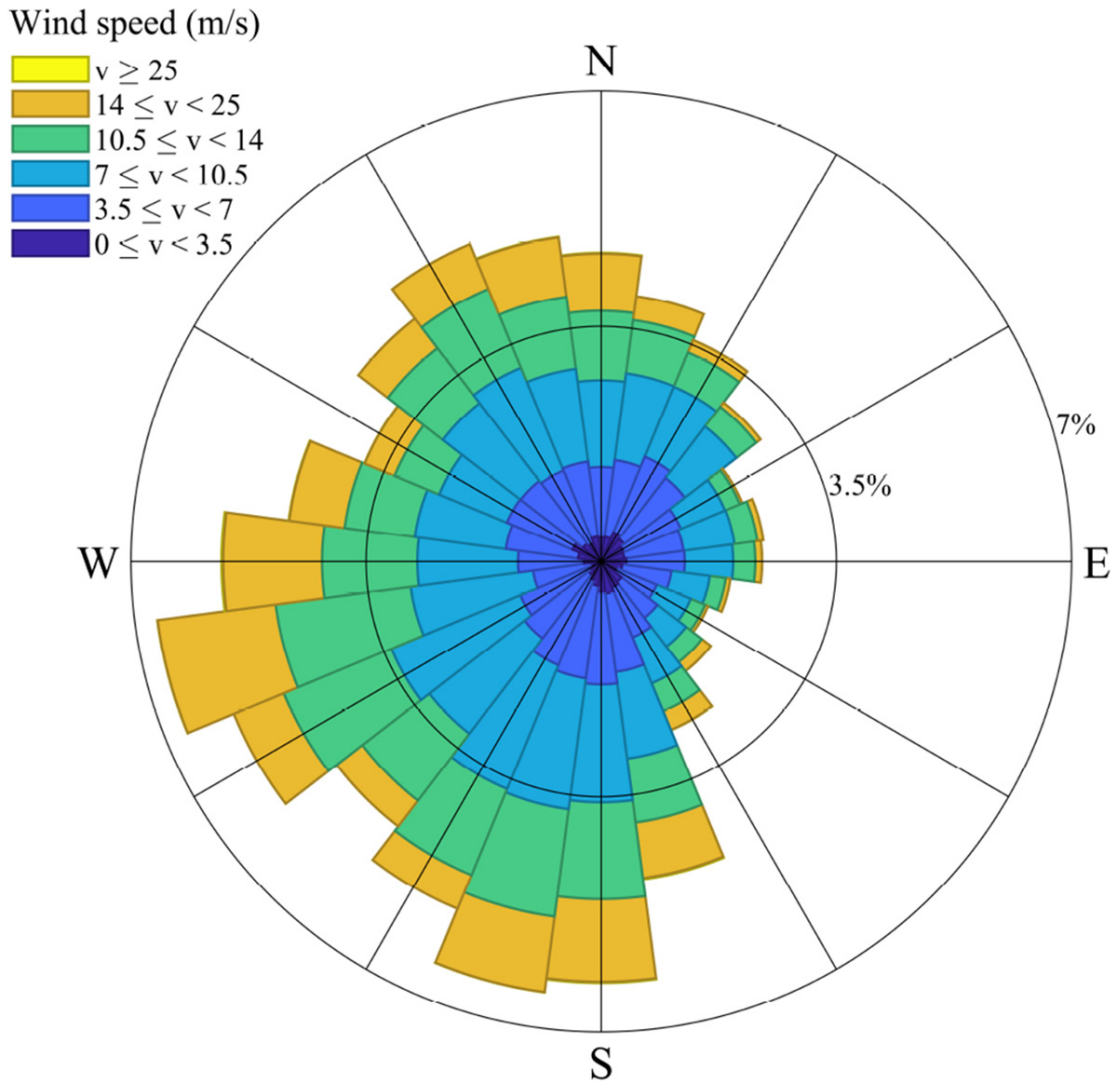
Wind Rose related to Table 4.

Although the CG optimization only needs the WTs’ nominal power, the MCA assessment requires the power curves to account for wind speed variations. Table 3 discloses information about the WTs of the studied OWPPs. The power curves are available in 38, 39, 40, 41. In the four OWPPs, *v _ci_* and *v _co_* are equal to 3.5 and 25 m/s. This work utilized polynomial fitting to obtain functions representing the power curves. The implementation is available in 32. The wind data in this paper comes from 42 and concerns real measurements from the Anholt OWPP LiDAR during 2013 and 2014. It contains speed and direction data averaged for every 10-minute interval. In addition, there is information about the availability of measurements at each interval. Across all data, intervals either with “Not a Number” values or with availability less than 20% were deleted. The latter were deemed as not being representative enough. Then, all data points were grouped into wind direction classes/bins with 15° range, which were fitted by Weibull functions ( Equation 16), known for adequately modeling wind distributions. Table 4 presents the obtained Weibull parameters. Figure 3 provides a Wind Rose representation created via 43. The *w _dir_* classes have different probabilities of occurrence ( *ρ*) according to the number of data points in each class. Note that the class 1 wind direction corresponds to a wind coming from the east.

$$\;f\left(v\right)=\frac{w_{1}}{w_{2}}\cdot {\left(\frac{v}{w_{2}}\right)}^{\left(w_{1}-1\right)}\cdot \text{exp}\left(-{\left(\frac{v}{w_{2}}\right)}^{w_{1}}\right)\;(16)$$

**Table 3.** Wind turbine models in the studied OWPPs.

	WT model	*P _n_* [MW]	*R _D_* [m]
**ORM**	REpower 5M	5	126
**HR1**	Vestas V80/2000	2	80
**TH**	Vestas V90/3000	3	90
**LA**	Siemens Gamesa SWT-3.6-120	3.6	120

**Table 4.** Wind distributions based on data from 42.

Class	*θ _l_* [°]	*θ _u_* [°]	*w*_1_	*w*_2_	*ρ*
**1**	0	15	2.1199	8.1139	0.0239
**2**	15	30	2.2040	8.1929	0.0243
**3**	30	45	2.2816	7.5707	0.0227
**4**	45	60	2.2784	7.9942	0.0299
**5**	60	75	2.1842	8.5800	0.0357
**6**	75	90	2.3105	9.5937	0.0399
**7**	90	105	2.1860	11.0869	0.0460
**8**	105	120	2.2148	11.0324	0.0488
**9**	120	135	2.4973	10.7246	0.0511
**10**	135	150	2.3871	10.2361	0.0456
**11**	150	165	2.0106	9.7692	0.0382
**12**	165	180	2.2321	11.0227	0.0469
**13**	180	195	2.4085	12.4347	0.0565
**14**	195	210	2.9876	12.7650	0.0666
**15**	210	225	2.9918	11.1243	0.0592
**16**	225	240	2.7971	10.3396	0.0496
**17**	240	255	2.7111	10.2746	0.0559
**18**	255	270	2.4548	11.1926	0.0646
**19**	270	285	2.2207	11.2526	0.0626
**20**	285	300	2.0183	10.7190	0.0476
**21**	300	315	1.8658	9.1812	0.0271
**22**	315	330	1.7994	8.4042	0.0206
**23**	330	345	2.2785	8.1085	0.0172
**24**	345	360	2.1086	7.8493	0.0194

**Figure 4.**
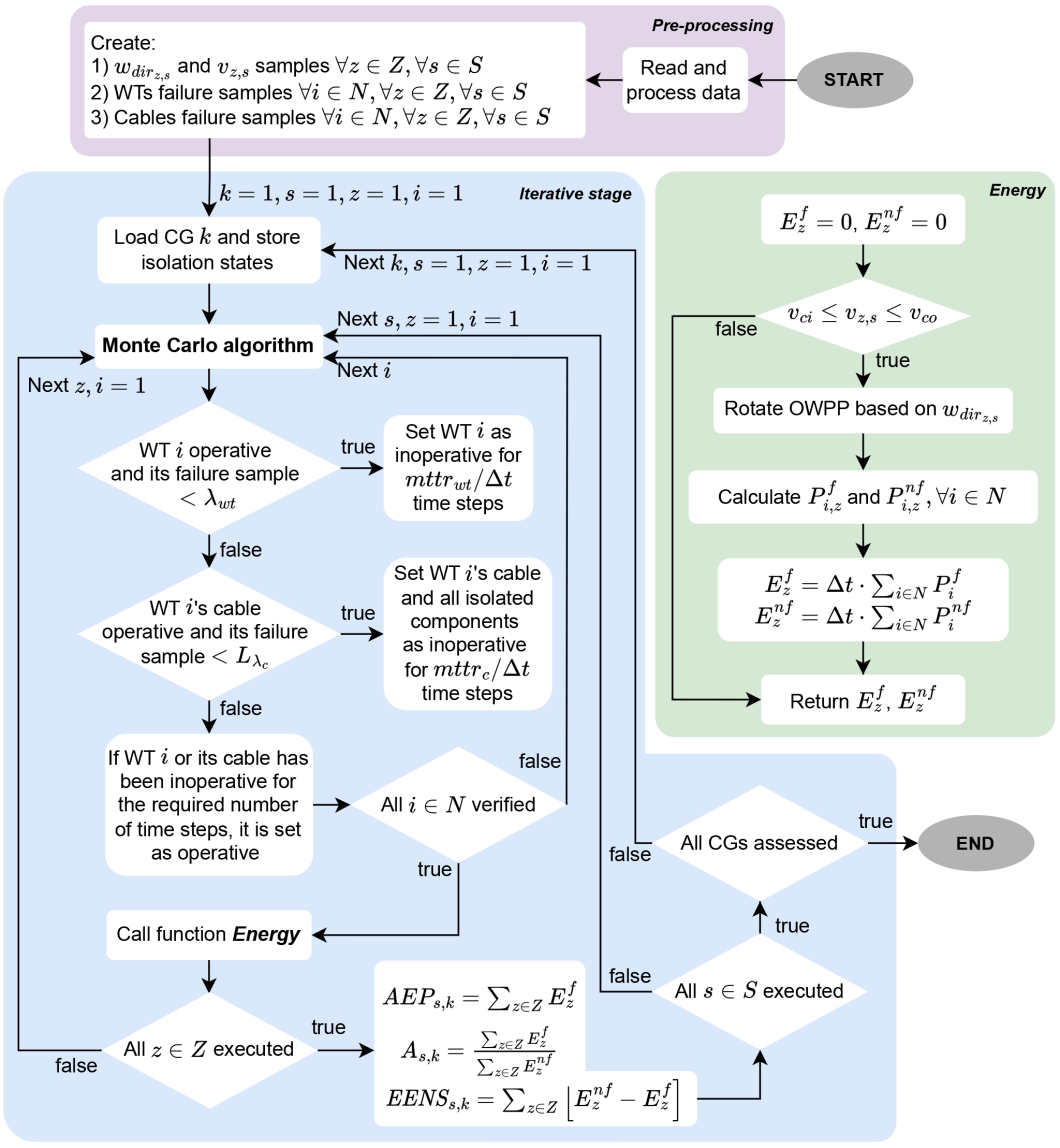
Flowchart of the MCA-based assessments.

Figure 4 shows a flowchart of the MCA for *EENS* estimation. Ahead are important remarks about the methodology.

**Figure 5.**
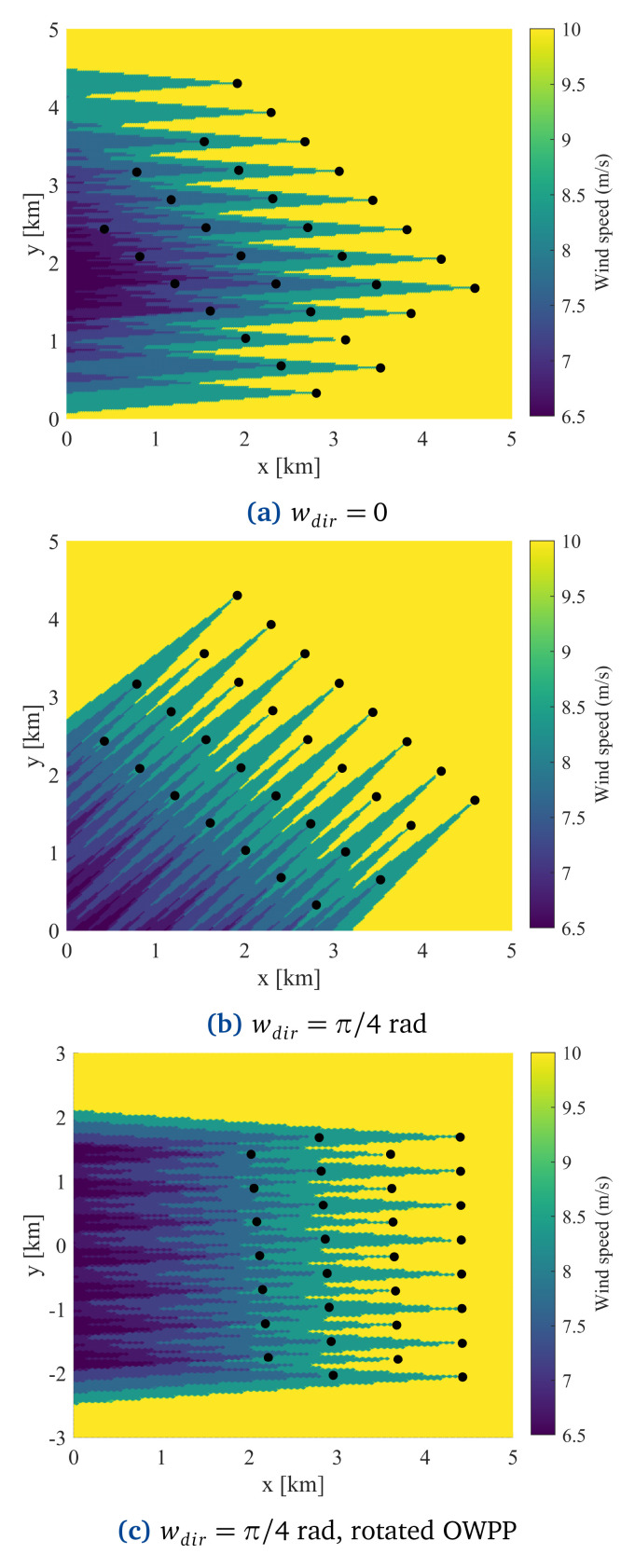
Wake effects illustration in the Ormonde OWPP based on Jensen’s velocity deficit model.

- At the beginning of the pre-processing stage, all OWPP data are read: WTs and substations coordinates, wind information, reliability data (failure rates and MTTR), and information about the WT model. Parameter *λ _c_* is given in occurrences per year per kilometer. The multiplication of *L* (from Equation 2) by *λ _c_* yields *L _λ _c__*, which provides the failure rates in occurrences per year for each potential cable installation in the OWPP. The yearly failure rates *λ _wt_* and *L _λ _c__* are adjusted to ∆ *t*.
- As a final step in the pre-processing stage, a quasirandom procedure creates *w _dir_* samples for every time step of every simulation. For each *w _dir _z,s__* sample, a *v _z,s_* value is randomized according to the related Weibull distribution in Table 4. Furthermore, the method creates WT and cable failure samples for every WT at every time step of every simulation. For instance, Ormonde has 30 WTs. Since this paper applies 1000 simulations with 1095 time steps each, there are 32850000 samples to indicate a potential WT failure over the entire assessment. All samples are real numbers ∈ [0, 1]. The class of a particular *w _dir _z,s__* sample comes from analyzing it against the probabilities *ρ* in Table 4.
- Upon loading a CG, all WTs and cables are operative in the first time step. At each time step of each simulation, if a failure sample is less than the ∆ *t*-adjusted failure rate and the component (WT or cable) is operative, the component is set as inoperative for the required number of time steps according to the MTTR. For WTs, the failure sample is promptly compared against *λ _wt_*, whereas failure samples of cables are compared with *L _λ _c__*. A cable failure isolates all downstream WTs and cables in a radial grid. Hence, when a cable experiences failures, all downstream components are set as unavailable following the MTTR of the faulty cable. This paper obtains an undirected graph from the connection matrix *u′* (from Equation 11) and then utilizes graph theory to detect isolated components ( Graphs package, version 1.9.0). After the MTTR-related number of time steps following a failure, relevant components are set again as operative.
- At each time step of the iterative stage, Figure 4’s function *Energy* quantifies the energy output of the WTs based on the wind condition. A class 1 *w _dir_* corresponds to an east wind. For other classes, Equation 17 rotates the OWPP clockwise so that the wind comes from the east in the transformed system, as illustrated in Figure 5. Note that the *w _dir_* value is taken as the center of the class/bin range. The rotation procedure simplifies wake calculations. For clarification, when a WT extracts energy from the wind, the airflow is affected, and the wind speed decreases behind the rotor, which impacts the power output of WTs receiving the same airflow. This phenomenon relates to the so-called wake effects ^44^.
$$\left[\begin{array}{c} x_{j}^{\prime }\\ j_{j}^{\prime } \end{array}\right]=\left[\begin{array}{cc} \text{cos}\left(w_{dir}\right) & \text{sin}\;\left(w_{dir}\right)\\ -\text{sin}\left(w_{dir}\right) & \text{cos}\;\left(w_{dir}\right) \end{array}\right]\cdot \left[\begin{array}{c} x_{j}\\ y_{j} \end{array}\right],\forall j\in N_{0}\;(17)$$
- This paper uses Jensen’s model ^45, 46^ to estimate wake effects. Readers interested in an implementation-oriented formulation of the model are referred to 47. Jensen’s model requires the decay constant ( *d _c_*) and thrust coefficient ( *t _c_*), which are sites and WT-dependent quantities not trivial to obtain. For simplification, this paper considers values equal to 0.094 and 0.88, respectively ^47^. The wake calculations in this work consider the failure states of the components since inoperative WTs do not affect the operative WTs. However, it is also needed to calculate wake neglecting failures to estimate the unavailable energy. After quantifying wakes, the generated power comes from the WT power curve according to the wake-adjusted wind speed.
- After sweeping all time steps, one can calculate the current CG’s *EENS*, *A*, and *AEP* in each simulation. Upon execution of all simulations, Equation 18, Equation 19, and Equation 20 yield the mean values of the mentioned variables.
$$\;{\bar{EENS}}_{k}={\left|S\right|}^{-1}\cdot \sum_{s\in S}EENS_{s,k}\;(18)$$
$$\;{\bar{A}}_{k}={\left|S\right|}^{-1}\cdot \sum_{s\in S}A_{s,k}\;(19)$$
$$\;{\bar{AEP}}_{k}={\left|S\right|}^{-1}\cdot \sum_{s\in S}AEP_{s,k}\;(20)$$

## 4 Results and discussions

This section provides and discusses the results from the cabling optimizations and the Monte Carlo simulations. The whole study was done in a 64 GB RAM, Intel ^®^ Core™ i9-11950H workstation. The following subsections report the findings from each part of the research.

### 4.1 Inter-array cabling optimization

**Figure 6.**
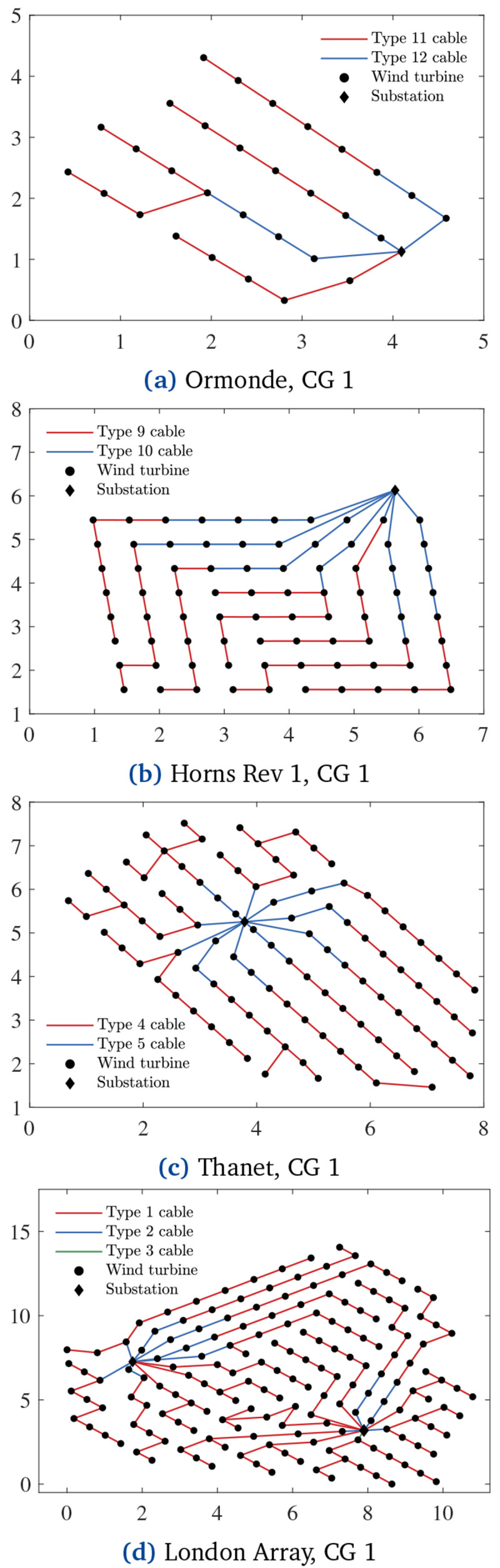
Cost-optimal CGs, x and y axes in [km].

**Figure 7.**
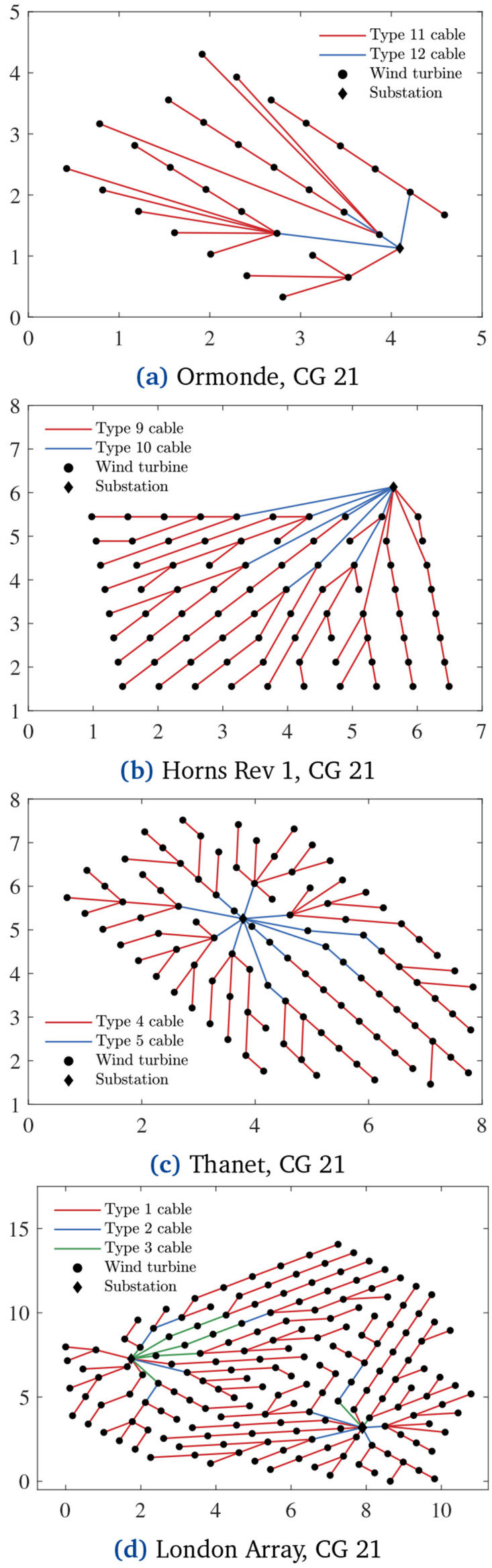
PCR-optimal CGs, x and y axes in [km].

The CG optimization approach described in subsection 3.1 starts by obtaining the normalization factors for Equation 14 through the decoupled minimization of costs and PCR. Table 5 provides the results for these cases. Figure 6 and Figure 7 show the layouts related to the separate cost and PCR minimization, respectively.

**Table 5.** Results for the exclusive cost [M€] and PCR [MWkm] minimizations.

	ORM		HR1		TH		LA	
	Cost	PCR	Cost	PCR	Cost	PCR	Cost	PCR
**Cost minimization**	8.0821	74.0577	22.5769	340.0923	26.6447	278.2256	61.2416	747.5269
**PCR minimization**	13.7739	68.5635	32.7151	290.6065	33.1062	249.1900	76.2046	678.9465

A few observations related to the exclusive cost minimization in each OWPP:

- A separate optimization with no heuristics confirmed the global optimality of the M€ 8.0821 Ormonde cost. This cost is slightly above the one in 18. This is probably related to small deviations in the x and y coordinates of the OWPP elements. As a reminder, all optimizations in this paper had the optimality gap set to zero.
- The cost of M€ 22.5769 obtained for Horns Rev 1 is slightly below the one in 18. Once more, small deviations in the x and y coordinates are probably the reason for the difference in results. The Horns Rev 1 optimization was initially combined with the presented heuristic, meaning that its global solution could have been compromised. However, the full problem, i.e., without heuristics, was optimized with a gap tolerance of zero in a separate test by taking the cost-minimization solution in Table 5 as initial guesses for the decision variables of the full optimization problem. After 12 minutes, CPLEX confirmed its global optimality.
- The cost of M€ 26.6447 obtained for Thanet matches the one in 18. Analogously to the Horns Rev 1 case, CPLEX confirmed its global optimality 1 hour and 29 minutes after taking Table 5’s solution as initial guesses for the decision variables in the full problem.
- The cost of M€ 61.2416 obtained for London Array is less than the one in 18. However, this comparison is not meaningful since, for this OWPP, reference 18 considered power loss costs on top of the ones in Table 1. Reference 18 presented a power loss costs estimation strategy that precedes the optimization, hence imposing no additional computational burden related to additions of loss calculation constraints. However, the London Array loss costs were not disclosed in 18. In addition, the authors of this paper could not find the technical information of the cables to enable replication of such costs. Thus, this paper optimized London Array accounting exclusively for purchase and installation costs. There was no success in optimizing the full London Array optimization problem with initial guesses given by the solution in Table 5. Due to the size of the OWPP, the optimization model becomes significantly heavy. After 3 hours and 40 minutes, CPLEX interrupted the optimization attempt due to running out of memory. Thus, the authors cannot state any information about the true optimality gap of the M€ 61.2416 cost.

Figure 7 demonstrates how PCR affects the CGs. Compared with the cost-optimal CGs in Figure 6, the ones in Figure 7 have more branches due to the inclusion of PCR in the objective function. In other words, Figure 7’s CGs are less prone to energy losses when cable failures occur.

**Figure 8.**
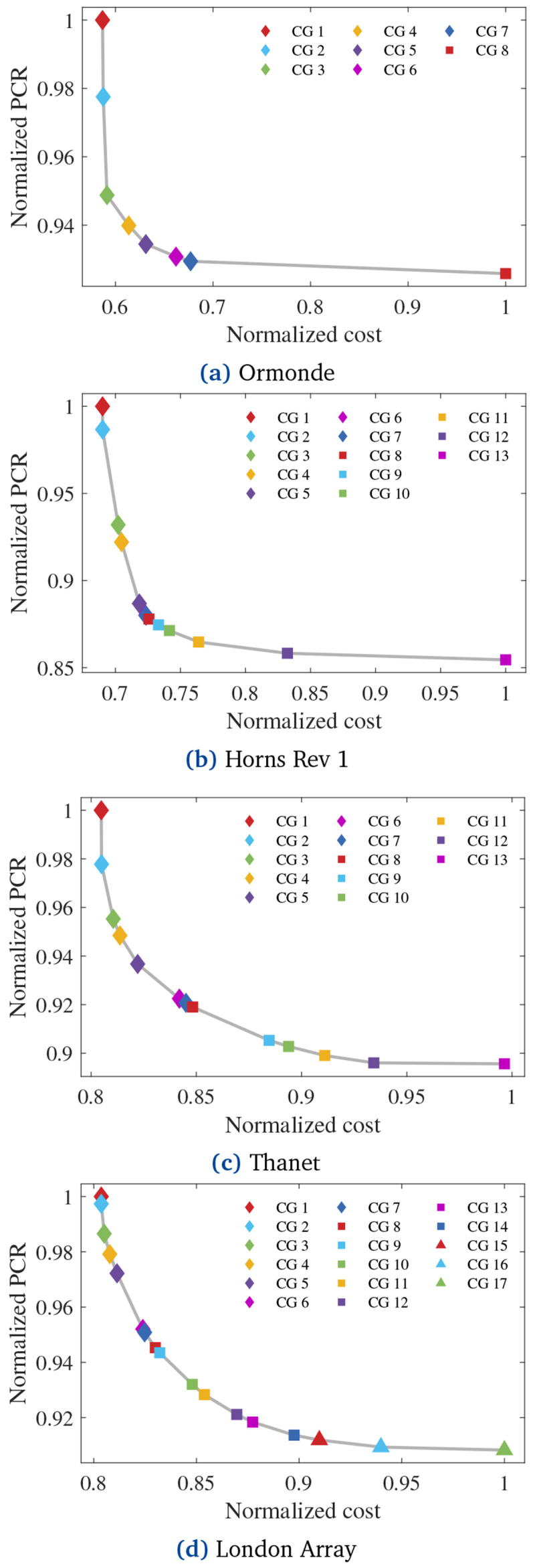
Pareto solutions.

As a reminder, in each OWPP, Equation 14’s normalization factor for ℱ _1_ comes from the cost obtained in the PCR minimization. In contrast, the normalization factor for ℱ _2_ regards the PCR obtained in the cost minimization. To analyze the criteria trade-offs, 21 optimization cases for each OWPP decrease from 1 to 0 with a step of 0.05. All cases were minimized according to the objective function in Equation 14 subjected to the constraints in Equation 3 to Equation 10 and Equation 12. As seen in Table 6, some optimization cases converged to the same CG. Figure 8 shows the Pareto solutions.

**Table 6.** Optimization cases and resulting CGs.

ORM cases	HR1 cases	TH cases	LA cases	CG
1	1	1	1	1
2,3	2,3,4	2,3,4	2,3	2
4-15	5	5,6,7	4,5,6	3
16	6	8,9	7	4
17,18	7,8,9	10,11,12	8	5
19	10	13	9	6
20	11	14	10	7
21	12,13,14	15	11	8
	15	16	12	9
	16	17	13	10
	17,18,19	18	14	11
	20	19,20	15	12
	21	21	16,17	13
			18	14
			19	15
			20	16
			21	17

**Figure 9.**
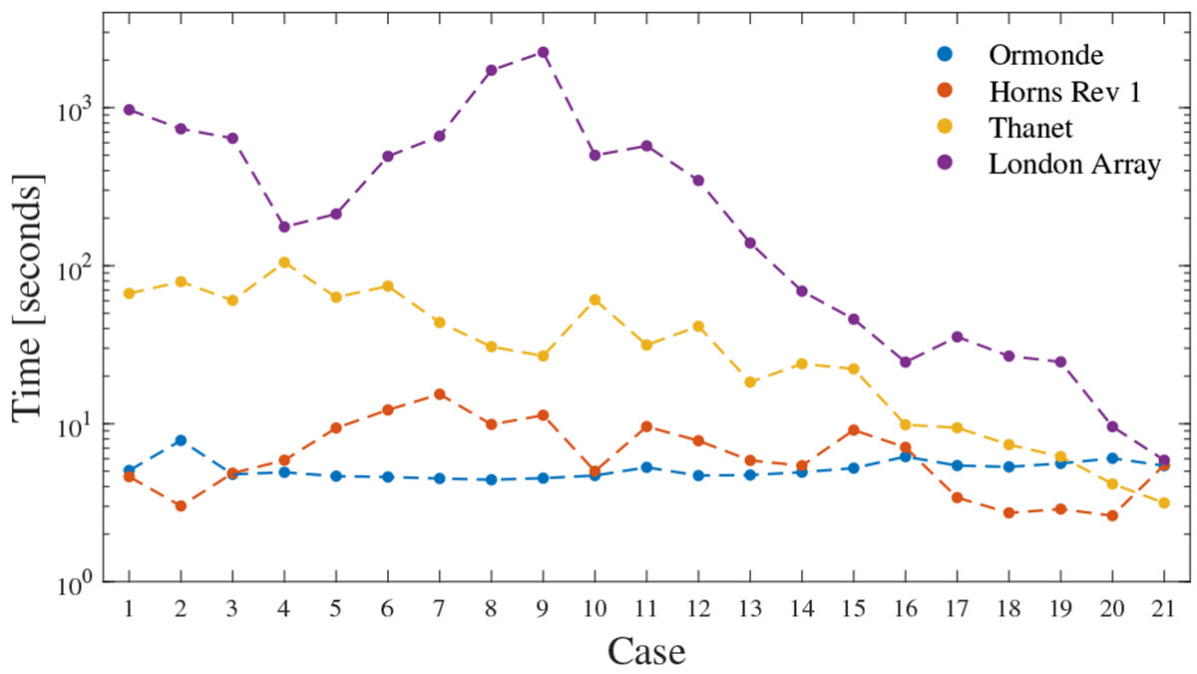
Optimization duration.

Regarding computational time, Figure 9 shows the optimization duration in each OWPP. The Ormonde and Horns Rev 1 cases required *≈* 5 to 8 and 3 to 20 seconds, respectively. Most of the Thanet cases required more than 10 seconds, up to *≈* 100 seconds. Most of the London Array cases demanded more than 100 seconds, with the longest lasting for *≈* 2500 seconds. The Thanet and London Array cases suggest faster convergence when the PCR criterion outweighs the cost criterion. Although the optimization heuristic is not the focus of this paper, it is interesting that the cost minimization in 18 for Horns Rev 1, Thanet, and London Array required 1 minute, 107 minutes, and 748 minutes, respectively. In this paper, such cases took 5.1 seconds, 4.6 seconds, and 17 minutes, respectively.

The next subsection addresses the relation between PCR and *EENS*. Consequently, the potential of the proposed PCR to indicate the reliability of radial CGs becomes clear.

### 4.2 Monte Carlo-based reliability assessment

**Figure 10.**
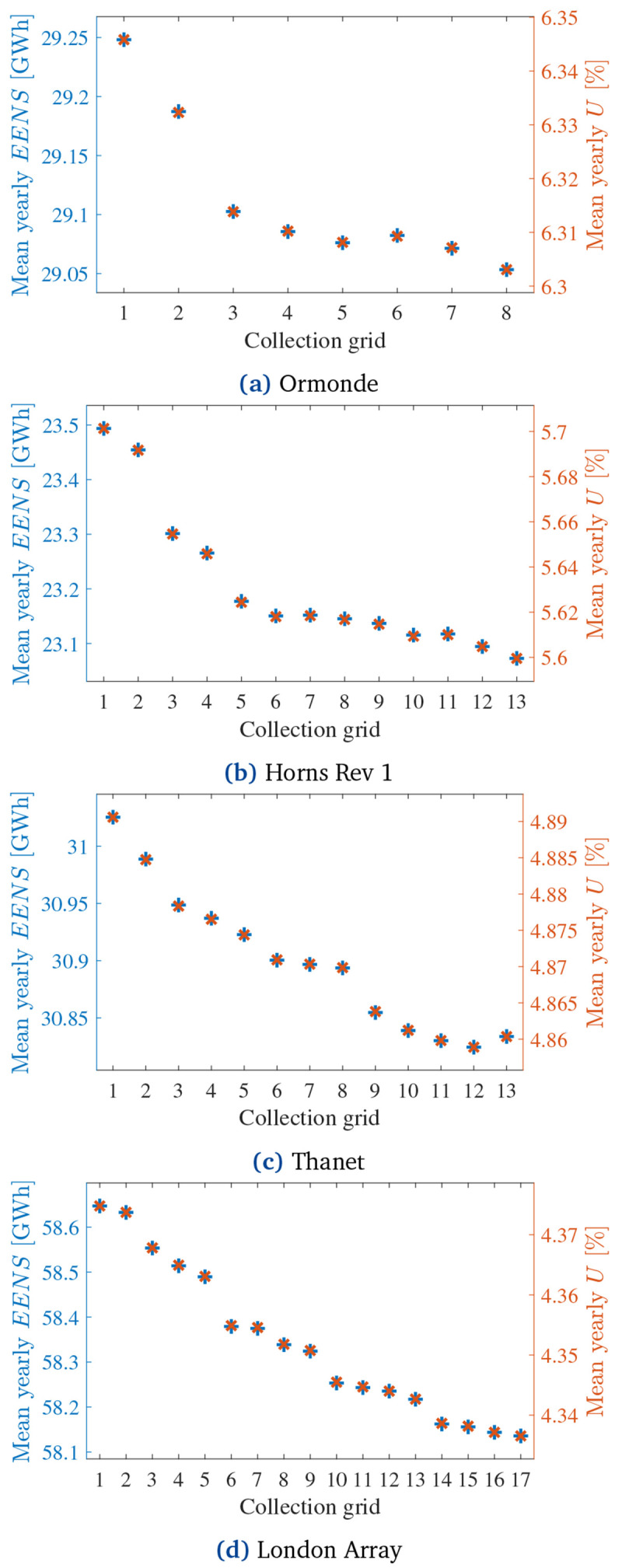
$\bar{\text{EENS}}$ and *Ū* of all CGs.

**Figure 11.**
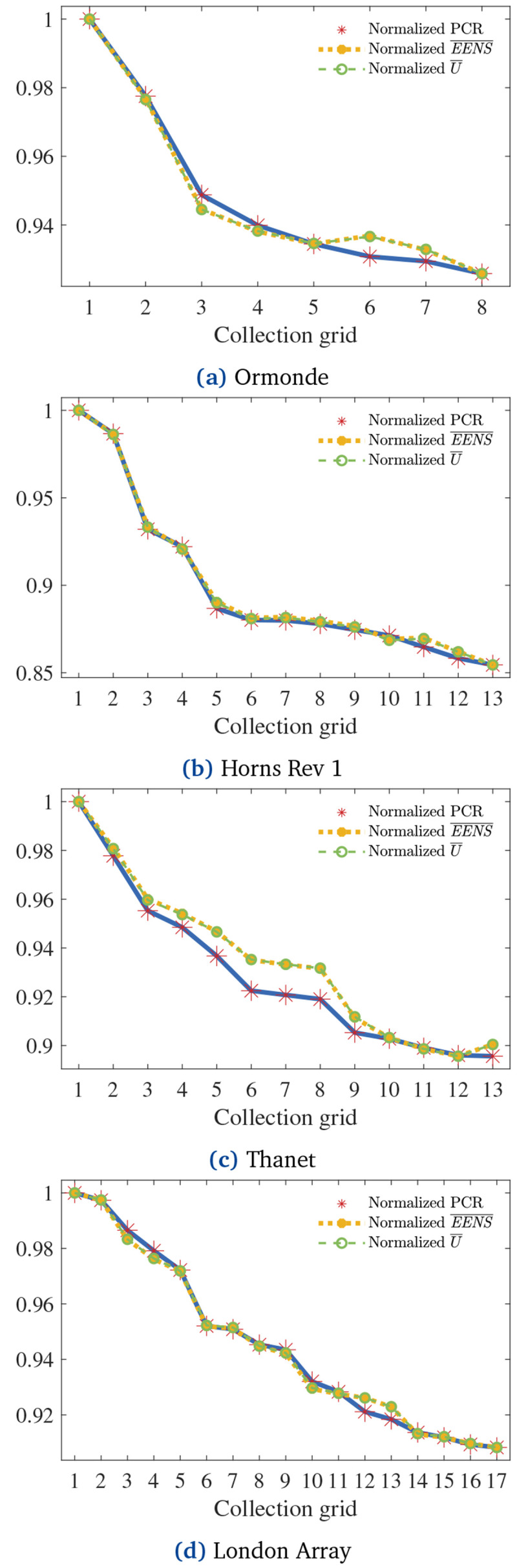
$\bar{\text{EENS}}$ and *Ū* normalized according to PCR.

The quasi-random MCA from subsection 3.2 assessed the $\bar{EENS}$ and *Ā* of all CGs from Figure 8 according to 1000 yearly simulations with time steps of 8 hours. Figure 10 presents the obtained yearly $\bar{EENS}$ and mean unavailability ( *Ū* = 1 *− Ā*) curves. Figure 11 normalizes $\bar{EENS}$ and *Ū* regarding the normalized PCR of each CG in each OWPP.

Visual inspection of Figure 11 reveals strong correlations between $\bar{EENS}$ and the normalized PCR. Pearson’s correlation coefficients between the curves for Ormonde, Horns Rev 1, Thanet, and London Array are 0.9934, 0.9992, 0.9885, and 0.9979, respectively. Such results suggest that the proposed PCR effectively indicates the reliability of the layouts when optimizing radial CGs under the assumptions made. As remarks: i) a normalized PCR equal to 1 relates to the *EENS* of the cost-optimal radial CG; ii) the PCR calculation does not enable the quantification of the ratio between the *EENS* of a certain CG and the cost-optimal *EENS* ( *EENS _CO_*). For instance, if the normalized PCR of a non-cost-optimal CG is equal to *β* ∈ [0, 1], it is false that ^*EENS*^/ _*EENS _CO_*_ = *β*. Observation ii reveals the weakness of the PCR approach. Still, the obtained results indicate that addressing PCR strongly correlates with improving the expected reliability of a radial CG.

**Figure 12.**
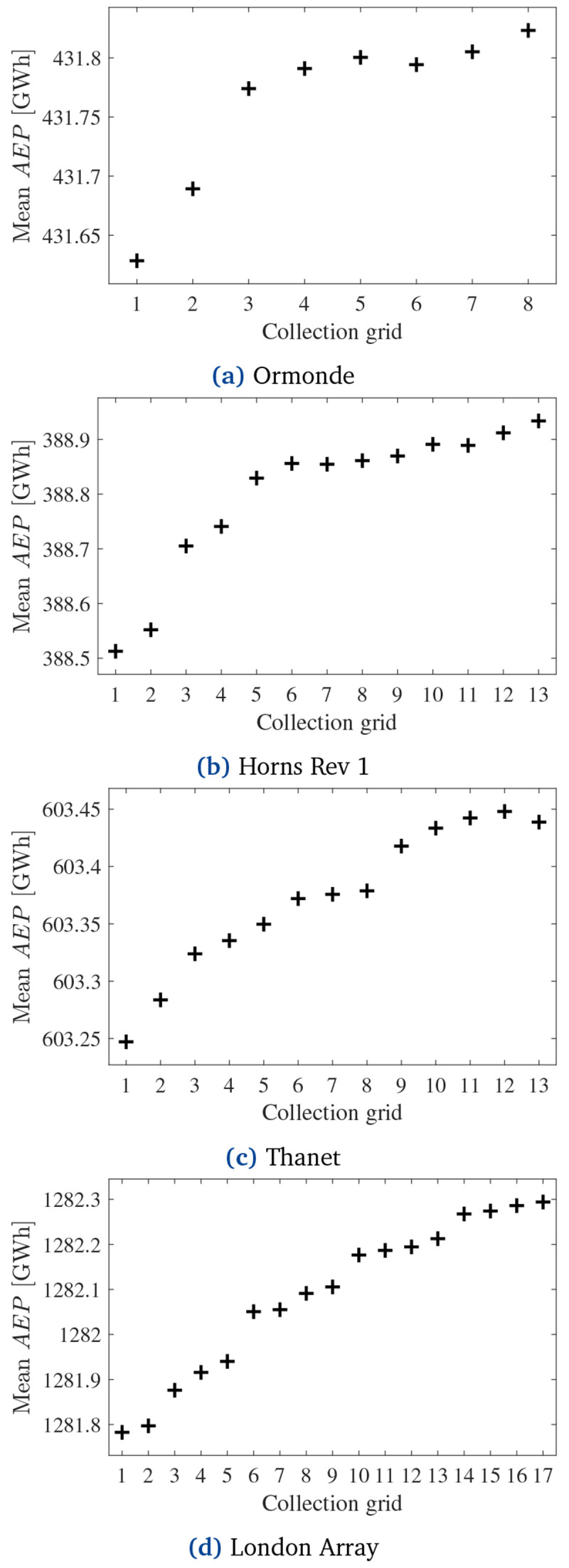
Mean *AEP* of all CGs.

Despite the discussions in the previous paragraph, it is important to note that a more reliable CG, i.e., one that tends to deliver a higher *AEP*, is not necessarily the most profitable alternative. Installing a more reliable cabling layout and increasing the energy supply comes with a higher initial investment into the CG. Thus, Equation 21 evaluates the levelized cost of energy ( *LCOE _k_*) regarding the total investment (ℐ _*k*_) into the OWPP according to CG *k*. The yearly *AEP* is given by the mean (expected) production of each CG, also calculated by the MCA ( Equation 20) and shown in Figure 12. Based on 7, 8, this paper considers the cable investment of the cost-optimal CG (ℱ _1 _1__, given by the cost values in the first row of Table 5) to be 12.5% of the total OWPP costs, meaning that the non-cabling costs are equivalent to 7 times ℱ _1 _1__. Therefore, for all CGs from Figure 8, Equation 22 quantifies ℐ _*k*_. The discount rate and OWPP lifetime for Equation 21 were taken as 0.07 and 25 years.

$$\;LCOE_{k}=\frac{I_{k}}{\Sigma_{i=1}^{N_{y}}{\bar{AEP}}_{k}/{\left(1+d_{r}\right)}^{i}}\;(21)$$

$$\;I_{k}={ℱ}_{1_{k}}+7{ℱ}_{1_{1}}\;(22)$$

**Figure 13.**
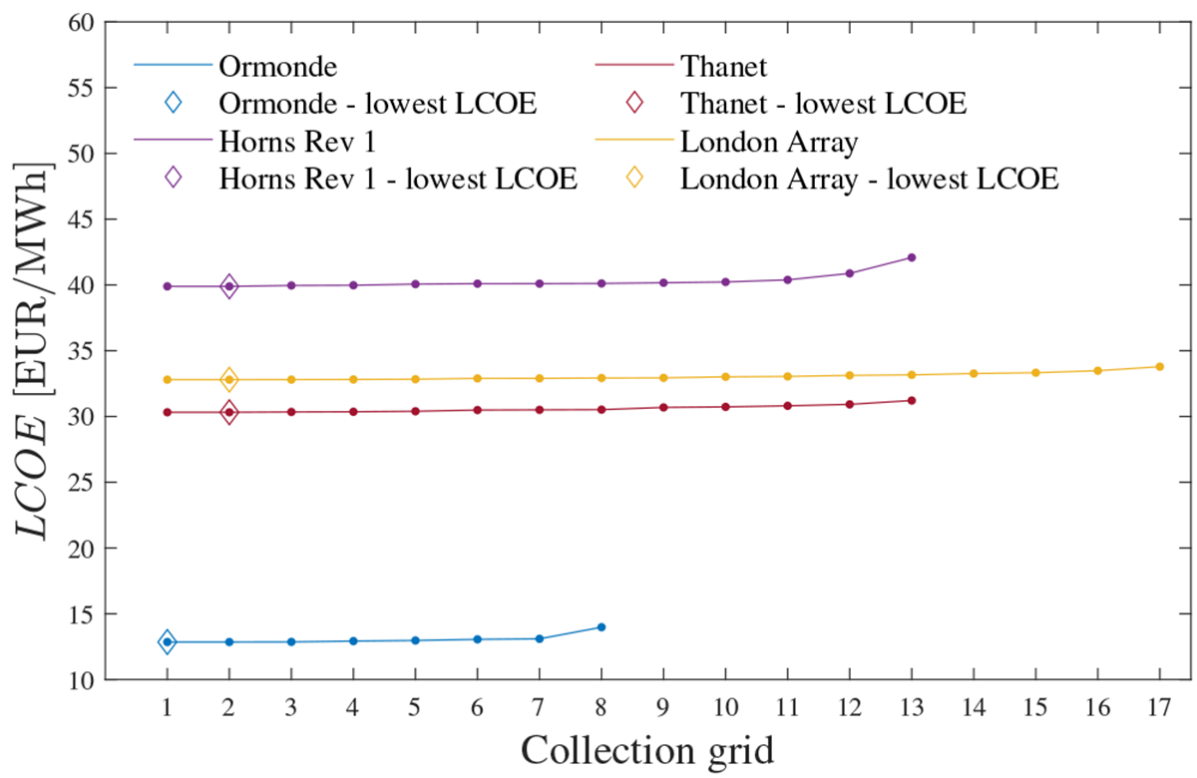
*LCOE* of the obtained CGs for each OWPP.

Figure 13 presents the *LCOE* of all optimized CGs. Concerning Ormonde, CG 1 ( Figure 6a) presented the lowest *LCOE*, indicating that additional investment into reliability is not cost-effective. In contrast, CG 2 in each of the other OWPPs yielded the lowest *LCOE*.

**Figure 14.**
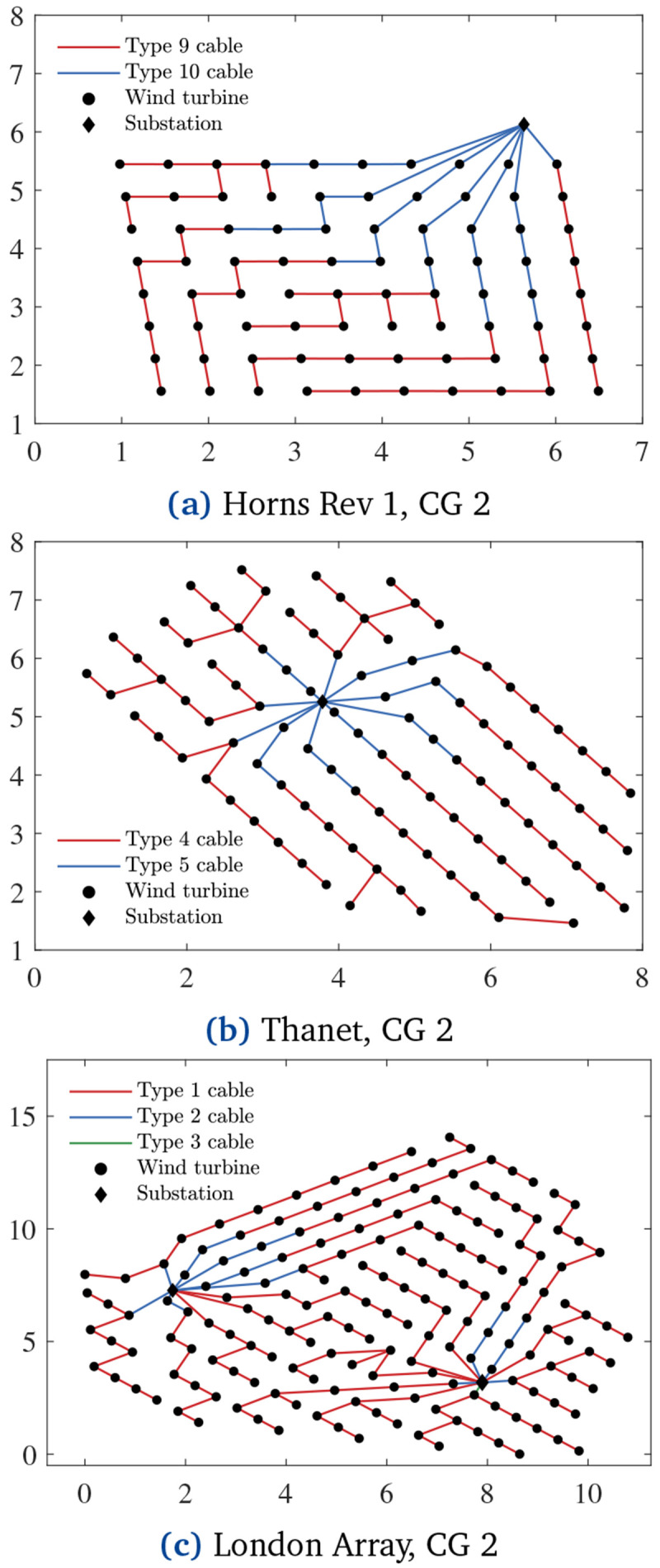
Lowest- *LCOE* CGs, x and y axes in [km].

Figure 14 shows CG 2 regarding Horns Rev 1, Thanet and London Array. The purchase and installation costs of these CGs are equal to M€ 22.5785, M€ 26.6491, and M€ 61.2420, respectively. These values imply increments of 0.0009%, 0.0021%, and 0.0001% in total OWPP investment ( Equation 22) against 0.0101%, 0.0061%, and 0.0011% expected additional energy supply compared with the cost-optimal CGs from Figure 6.

At this point, it is crucial to highlight the major assumptions made during the development of this paper:

- As described in the third paragraph of subsection 3.2, failure rates and MTTR came from the literature. The utilized values may not fully represent the failure probabilities and maintenance time of the WT and cable models in the assessed OWPPs.
- Despite relating to real measurements, the utilized wind data comes from the Anholt OWPP, meaning that the actual wind profiles in the four studied OWPPs differ from the one in this paper.
- Jensen’s model estimates wake losses in all OWPPs. Gao *et al.* ^48^ claims that this model tends to underestimate wind velocity deficits, hence overestimating power generation.

The mentioned assumptions affect the reported results. In real assessments, the utilized failure and wind data should be site-dependent. Therefore, upon utilization of information that better represents the studied OWPPs (which the authors were unable to obtain), the CGs with the lowest *LCOE* may differ from the ones in Figure 13. However, this work’s main goal is to demonstrate that the proposed PCR strongly correlates with *EENS* and that the presented optimization approach combined with Monte Carlo simulations can effectively indicate radial CGs that promisingly balance initial investments with reliability.

**Figure 15.**
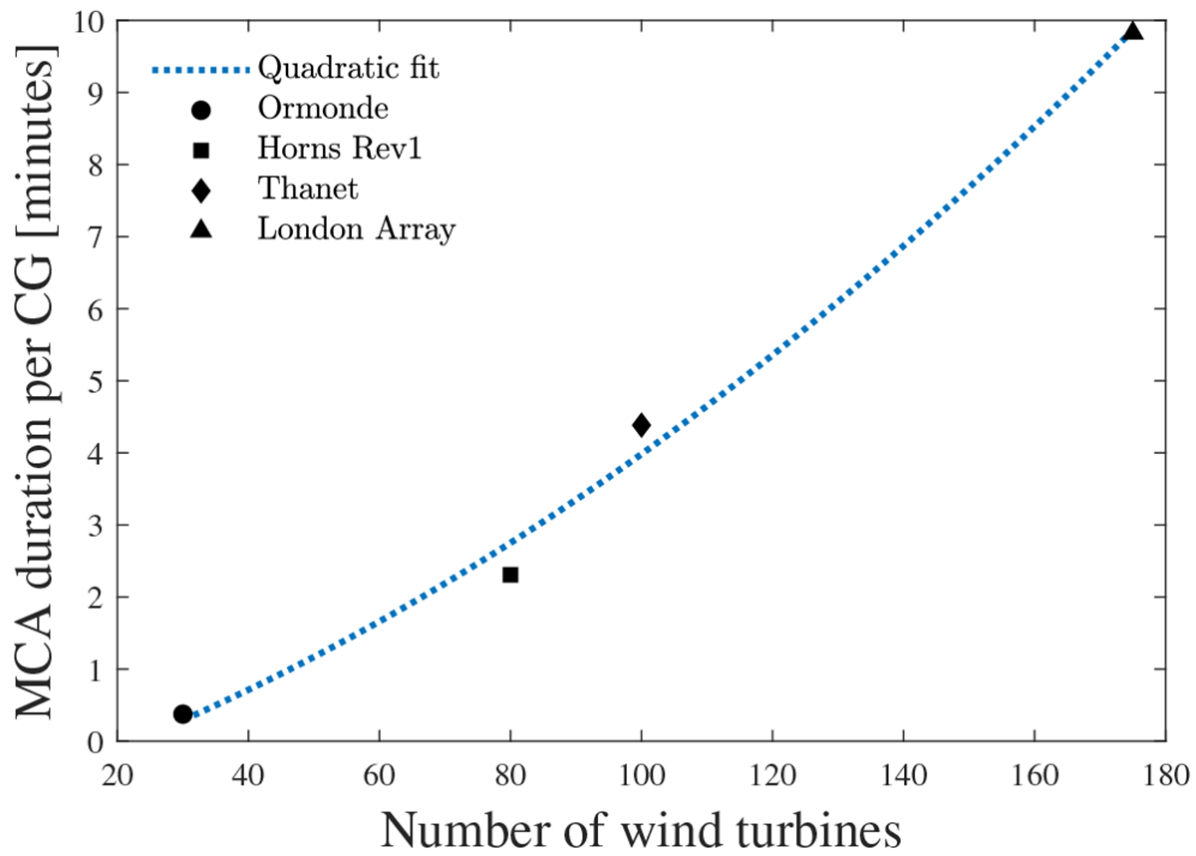
MCA computational performance.

Concerning the MCA computational performance, the implemented code required 3, 30, 57, and 167 minutes for the Ormonde, Horns Rev 1, Thanet, and London Array assessments. Figure 15 plots the required time according to the number of assessed CGs in each OWPP. This figure indicates that the assessment duration shows a somewhat quadratic behavior according to the OWPP size.

This work utilized 1000 yearly simulations with time steps of 8 hours. Note that increasing the former or decreasing the latter would cause the computational time to increase. Analogously, decreasing the former or increasing the latter would cause the computational time to decrease. The code was optimized for performance by considering Julia’s column-major array ordering. Furthermore, given the vast quantity of mathematical operations, the code leverages memory pre-allocation to minimize memory usage and enable faster computations. Even for the significantly large London Array OWPP, the MCA execution required less than 3 hours. Given that the addressed problem relates to the planning of assets meant to last for decades, the authors deem such simulation lengths as viable.

### 4.3 Potential addition of PCR to joint layout and CG optimization methods

In the context of OWPPs, *layout optimization* refers to establishing the optimal location (coordinates) of the WTs and substations within a pre-established area. The importance of adequately placing the components comes from different layouts implying different wake losses, which should be minimized.

**Figure 16.**
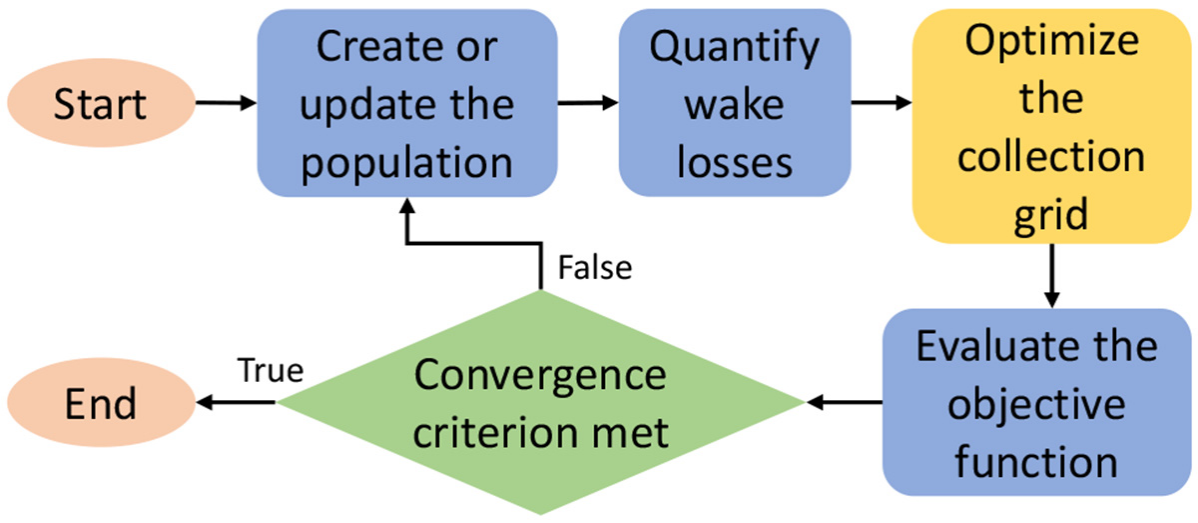
Basic structure of a joint layout and collection grid optimization framework.

Historical wind measurements, obtained from sources with meteorological expertise, can capture the wind speed and direction patterns in a particular site. Given the known wind behavior, experts utilize wake models to simulate the operation of a specific layout, thus estimating the energy that would be lost due to wake effects over a certain period. Metaheuristics are popular methods to address layout optimization problems ^44, 47, 49^. In such approaches, an individual of the population represents a specific layout, which “evolves” over the iterations. The literature presents papers that address layout optimization either as a standalone problem, such as 49, or jointly with cabling optimization, such as 47. In the latter, the CG is optimized before evaluating a specific individual (layout), and its cost is incorporated into the objective function. Figure 16 illustrates an overview of this approach.

The results from this paper indicate that the proposed PCR can add value to the described joint methods. By including the PCR criterion in the optimization model, joint methodologies could account for the reliability aspects of radial grids. However, potential computational burden issues should be considered.

As presented in subsection 2.2, adding the proposed PCR to the mathematical formulation is computationally light given the simple affine equations. However, depending on the number of WTs and the optimality gap, the overall CG optimization can be computationally heavy due to the binary variables, regardless of PCR. The following analysis takes Figure 16 as reference. In a metaheuristic approach with 30 individuals subjected to 100 iterations (i.e., 3000 evaluations), around 6.7, 7.5, 67, and 850 hours would be added to the joint layout and inter-array cabling optimizations of Ormonde, Horns Rev 1, Thanet and London Array, respectively. To a certain extent, this duration is acceptable for the first three OWPPs since the planning problem is not time-sensitive. Nonetheless, computational time can become an issue when addressing significantly larger OWPPs, such as the case of London Array. Computational burden problems could be alleviated by making the CG optimization heuristic more aggressive or relaxing the optimality gap, although both approaches can compromise high-quality solutions.

## 5 Conclusions

This paper presented a novel MILP approach to address the OWPP reliability regarding radial collection grids. The formulation considered technical constraints such as the cable-crossing prohibition and flow conservation. One of the optimization criteria came from the purchase and installation costs. The other concerned the proposed PCR, which sought to reflect the impact of cable failures. Case studies considering Ormonde, Horns Rev 1, Thanet, and London Array presented the associated Pareto solutions. A quasi-random MCA estimated the energy production and showed that, for the studied OWPPs, the widely utilized *EENS* index strongly correlates with PCR, which is the most significant contribution of this paper. Under the considered assumptions, the combined optimization and MCA methodology indicated that Horns Rev 1, Thanet, and London Array would benefit from additional cabling investments to decrease the LCOE, compared with the solution that minimizes purchase and installation costs.

As a highlight, the presented methodology enables the analysis of multiple cabling possibilities for the same OWPP. Such alternatives imply distinct trade-offs between investments and expected reliability. In contrast, the literature, including the works in Section 1, typically does not address CG optimization following a multi-criteria approach.

As limitations, the utilized failure rates and MTTR were retrieved from the literature and may not strongly represent values that are coherent with the studied OWPPs. Analogously, the wind data used for wind energy estimations relates to the Anholt OWPP, i.e., it may not fully capture the wind behavior in the investigated OWPPs. Lastly, the implemented Jensen’s velocity deficit model may not accurately estimate wake losses. Addressing such aspects would impact the obtained results and could lead to indications of different CGs as the ones with the lowest LCOE. In future research, the authors intend to: i) assess results yielded by modeling wake effects with alternative deficit models; ii) include other cost sources in the formulation, such as maintenance and power losses; iii) seek real historical power generation, maintenance, and wind data of a single OWPP as an attempt to validate the proposed PCR against real records of energy not supplied; iv) carry out a comparative study regarding the investment cost and reliability of radial and ring CGs.

## Ethics and consent statement

Ethical approval and consent were not required.

## Data Availability

All data underlying the results are available as part of the article and no additional source data are required. Zenodo: Extended data for “Inter-array cabling optimization in offshore wind power plants, a new reliability indicator for radial grids”, https://doi.org/10.5281/zenodo.10072864 ^30^ https://doi.org/10.5281/zenodo.8413581 ^32^ Data are available under the terms of the Creative Commons Attribution 4.0 International license (CC-BY 4.0)

## Software availability

Archived source code at time of publication: https://www.doi.org/10.5281/zenodo.8413581 ^32^

License: Creative Commons Attribution 4.0 International
