# Supplementary material for: Inter-array cabling optimization in offshore wind power plants, a new reliability indicator for radial grids
Source: Open Res Eur. 2024 May 28;3:214. Originally published 2023 Dec 1. [Version 2] doi: 10.12688/openreseurope.16716.2 (PMC11440233; doi:10.12688/openreseurope.16716.2)
Supplement: Peer review history

# Reviewer report: Reviewer response for version 2

**DOI:** 10.21956/openreseurope.19107.r41001

**Published:** 2024-06-11

**Authors:** Miftahul Fikri

**Affiliations:** 1 PLN Institute of Technology, Kota Jakarta Barat,, Jakarta, Indonesia

Manuscript is now Acceptable.

Is the study design appropriate and does the work have academic merit?

Yes

Is the work clearly and accurately presented and does it cite the current literature?

Partly

If applicable, is the statistical analysis and its interpretation appropriate?

Partly

Are all the source data underlying the results available to ensure full reproducibility?

Yes

Are the conclusions drawn adequately supported by the results?

Yes

Are sufficient details of methods and analysis provided to allow replication by others?

Partly

Reviewer Expertise:

High Voltage; Cable Insulation;  Modelling & Optimization;

I confirm that I have read this submission and believe that I have an appropriate level of expertise to confirm that it is of an acceptable scientific standard.

# Reviewer report: Reviewer response for version 2

**DOI:** 10.21956/openreseurope.19107.r40999

**Published:** 2024-05-31

**Authors:** Georgios Fotis

**Affiliations:** 1 ASPETE—School of Pedagogical and Technological Education, Heraklion, Greece

I believe the manuscript can be indexed in its current form.

Is the study design appropriate and does the work have academic merit?

Partly

Is the work clearly and accurately presented and does it cite the current literature?

Partly

If applicable, is the statistical analysis and its interpretation appropriate?

Partly

Are all the source data underlying the results available to ensure full reproducibility?

Partly

Are the conclusions drawn adequately supported by the results?

No

Are sufficient details of methods and analysis provided to allow replication by others?

Partly

Reviewer Expertise:

Power Systems;Transmission;Distribution;RES

I confirm that I have read this submission and believe that I have an appropriate level of expertise to confirm that it is of an acceptable scientific standard.

# Reviewer report: Reviewer response for version 1

**DOI:** 10.21956/openreseurope.18055.r39429

**Published:** 2024-05-03

**Authors:** Miftahul Fikri

**Affiliations:** 1 PLN Institute of Technology, Kota Jakarta Barat,, Jakarta, Indonesia

The topics and discussions are important research to be carried out currently and for future development. The study has also been presented from problems to solutions and conclusions obtained, but there are four main comments below so that the research can be improved:

1. Overall, the writing structure needs to be paid attention to in this manuscript because one section and another section form an inseparable storyline, and each has its role—likewise, the structure of the writing content in each section.
2. the author states two main contributions; this needs to be discussed logically, sequentially, step by step, and carefully so that the modeling results can be scientifically accepted.
3. The solution method (algorithm) and model validation must be adequately conveyed.
4. There are many inconsistent reference writings (superscript or not), symbols that need to be grouped, and typos.

In the Reviewer's view, the four comments above can be stated in each section (which is **optional**) for the Authors to follow. Following are the comments for each section:

 Section 1:

 The introduction presents the problems in OWWP, literature studies, contributions, and Discussions for Sections 2-6. However, this writing can still be improved by:

1. Pay attention to the structure of the writing (for example, deductive pattern writing) so that the OWWP problem will appear comprehensively by the topic, the research limitations, and the research contribution has a pattern like an inverted pyramid. Grouping main ideas between paragraphs can also help move the storyline in this introduction smoothly.
2. In the second contribution highlight, why doesn't the author write the proposed PCR as his contribution?
3. In the introduction, there is a statement: "An quasi-random MCA considering 1000 yearly simulations"?

Section 2: This is the most important discussion in this research because the author states that the highlight of the contribution is modeling improvement, so this discussion needs to be done carefully:

1. Modeling is intended so that the real OWWP problem can be represented in a mathematical equation with MILP to be analyzed further. Because the issue raised is quite complex, it is recommended that the author conduct this discussion point by point with certain groupings. This grouping can be done following the modeling pattern of references; [13]: sub-sections III.A, III.B, III.C;  [8] :  sub-sections 2.1, 2.2.1, 2.2.2, 2.3;   [10]: Sections 3 and 4;    [12]: Sub-sections II.A – II.G
2. Authors are advised to create a list of nomenclature such as variables, indexes, and sets that can refer to [10] and explain input, parameters, and output so that similar work can be replicated and developed for future research.
3. Section 2.1 is retained because it is a contribution made by the author.
4. There are still many inconsistent writing references (superscript or not)

Section3:

1. This section has no comprehensive algorithm for solving the model (only the EENS flowchart). This section should discuss the methodology for solving the model created in the previous section (Section 2); of course, Sections 3.1 and 3.2 are included in this comprehensive method/algorithm. This can be seen in Figure 2 and Algorithm 1 [8], Section 5 [10], algorithm [12], and others.
2. In completing the MILP, it is necessary to convey the method for solving it in this research, for example, branch & bound, Pareto optimal, breath-first search, or depth-first search.
3. Many symbols also appear in this section *λ* *c* *,* *λ* *wt* *,* *w* *dir* *,* *n* *wt* *, * etc.; of course, they need to be connected to the model in Section 2, likewise with equation (23).
4. There are still many inconsistent writing references (superscript or not).

Section 4:

1. it is recommended to be "results and discussion", which discusses the results of research conducted by researchers and discusses them in Section 4.1.
2. Then, Sub-Section 4.2 compares the results obtained (with PCR) and without including PCR so that validation and the impact of novelty are more visible.
3. Sub-Section 4.3 can be taken from 5.1.

Section 5:

1. Sub-section 5.1 is brief with CG ring, which should be included in sub-section 4.3.
2. sub-section 5.2 about potential PCR for future work should be included in the conclusion section (delivered after the contents of the conclusion are presented)

Section 6:

 Section 6 can become Section 5 because Section 5.1 is moved to Section 4, and Section 5.2 can be moved to this section and written after the conclusions made by the author are presented.

Is the study design appropriate and does the work have academic merit?

Yes

Is the work clearly and accurately presented and does it cite the current literature?

Partly

If applicable, is the statistical analysis and its interpretation appropriate?

Partly

Are all the source data underlying the results available to ensure full reproducibility?

Yes

Are the conclusions drawn adequately supported by the results?

Yes

Are sufficient details of methods and analysis provided to allow replication by others?

Partly

Reviewer Expertise:

High Voltage; Cable Insulation;  Modelling & Optimization;

I confirm that I have read this submission and believe that I have an appropriate level of expertise to confirm that it is of an acceptable scientific standard, however I have significant reservations, as outlined above.

# Response

**Published:** 2024-05-28

**Authors:** Ramon Abritta Aguiar Santos

Dear Dr. Miftahul Fikri,

 We (the authors) submitted the second version of the paper before your review report became available in Open Research Europe. However, as a fortunate coincidence, we believe this new version addresses many of your comments, which we recognize as highly pertinent and worthy of attention. We improved the structure of the paper so that the story was conveyed more smoothly, added a nomenclature table to facilitate the reading process, and addressed typos. We highlight that when we wrote "reliability indicator" in the second contribution (Section 1), we referred to PCR, although this has not been explicitly indicated. Furthermore, by "a quasi-random MCA considering 1000 yearly simulations", we mean that the MCA carries out 1000 assessments, each representing one year of operation. We have also mentioned the algorithms the solver utilizes to address the mathematical optimization problem. In addition to the previous statements, we have applied the methodology to larger (and real) power plants and sought to enrich the work by adding an LCOE analysis. Note that the ring CG analysis has been excluded since the utilized references do not provide ring CGs for comparison. Given the reasons above, we hope you have the availability to review this new version of the work. Thank you for dedicating part of your valuable time to our paper. We appreciate it. Regards,

 The authors

# Reviewer report: Reviewer response for version 1

**DOI:** 10.21956/openreseurope.18055.r39428

**Published:** 2024-04-18

**Authors:** Georgios Fotis

**Affiliations:** 1 ASPETE—School of Pedagogical and Technological Education, Heraklion, Greece

An approach to radial collection grid optimization is presented in this research to help offshore wind power project (OWPP) investment decisions. The suggested approach effectively integrates various optimization factors and facilitates the trade-off between cost and power capacity risk (PCR), which is characterized as a cumulative power flow summation that accounts for the effects of cable failures. The topic of this paper is interesting to the readers, however a great number of changes must be done:

1. The abstract has to be enhanced by explicitly stating the issue, outlining the goals of the study, outlining the methodology, providing specifics of the findings, and outlining the key conclusions.
2. The Introduction needs to be updated and made longer. To demonstrate the significance and originality of their work, the writers must, at the very least, present the current state-of-the-art to readers who are not familiar with the general research topic. The present references cited should be examined more closely, and many more relevant references should be included.
3. Highlight the unique features, advantages, or advancements of the methodology that distinguish it from other approaches in the field.
4. How does this effort advance the field in comparison to previously published works? Compare your findings with those of other researchers.
5. Make the conclusion part longer. It's far too short. Include the limitations of your study and recommendations for future work in the conclusion section.

Is the study design appropriate and does the work have academic merit?

Partly

Is the work clearly and accurately presented and does it cite the current literature?

Partly

If applicable, is the statistical analysis and its interpretation appropriate?

Partly

Are all the source data underlying the results available to ensure full reproducibility?

Partly

Are the conclusions drawn adequately supported by the results?

No

Are sufficient details of methods and analysis provided to allow replication by others?

Partly

Reviewer Expertise:

Power Systems;Transmission;Distribution;RES

I confirm that I have read this submission and believe that I have an appropriate level of expertise to confirm that it is of an acceptable scientific standard, however I have significant reservations, as outlined above.

# Response

**Published:** 2024-04-25

**Authors:** Ramon Abritta Aguiar Santos

Dear Dr. Georgios Fotis, We greatly appreciate your suggestions, which helped us significantly improve our work. Please note that we have detected a flaw in the methodology the way it was described in the first version of the manuscript. In summary, the proposed PCR needs to be weighed by the cable lengths. Otherwise, the metric will not correlate well with the *expected energy not supplied. *The flaw had gone unnoticed in the first version since its consequences are negligible in small wind power plants. We have substantially changed the paper to amend the mentioned flaw and make the method scalable for larger wind power plants. The formulation has been entirely replaced, and the description of the Monte Carlo-based assessment has been made more comprehensive. Here are comments addressing each item you included in your review report: (1) the abstract has been extended by following the provided guidelines; (2) the introduction has been expanded and now includes the new literature references 13, 14, 15, 19, and 21; (3) a new paragraph below the bullet points for contributions provides the reasons justifying the proposed approach, and distinguishing each from the literature. Highlighted aspects have also been summarized in the conclusions; (4) We could not directly compare reliability since this characteristic is site-dependent and data-dependent. Despite our efforts, we could not find works addressing the same wind power plants in similar conditions. However, the section 4.1 now features cost-related comparisons regarding work from the literature; (5) the conclusions have been extended as an attempt to make it more comprehensive. Limitations have been included (and also in the discussions on page 13). Thank you for your valuable suggestions. Regards, the authors.

# Reviewer report: Reviewer response for version 1

**DOI:** 10.21956/openreseurope.18055.r37745

**Published:** 2024-03-04

**Authors:** Xinwei Shen

**Affiliations:** 1 Tsinghua University, Beijing, Beijing, China

This paper presents a radial CG optimization method that combines investment cost and Power Capacity Risk (PCR). This is indeed a timely topic. However, the literature review should be improved, the model formulation is questionable, and the case studies are also not convincing enough. My concerns are listed below, which indicate the need for **substantial revisions** and improvements in the manuscript:

 1. The literature review in the manuscript appears to be insufficient. The authors need to conduct a more thorough literature review. The optimization of CGs from both economy efficiency and reliability perspectives has already been extensively investigated in prior works [1]-[4]. Therefore, I do not consider this aspect to be a significant contribution of the paper.

 2. It would be beneficial for the authors to clearly delineate how the candidate cables for the CG are determined.

 3. The proposed mathematical model lacks formulation on the power flow, which is crucial for the optimization of CGs. The authors should consider incorporating this aspect into the model.

 4. Why do the authors choose to use PCR as an indirect indicator of reliability rather than directly optimize the EENS as a reliability metric within the objective function? The authors should provide clarification regarding the motivation behind this approach.

 5. The proposed approach is applied to a particular test system, but it is still unclear how the solution can be scaled and extended to other systems. The scalability of the proposed planning method should be tested on some larger offshore wind farms to ascertain its applicability and effectiveness.

Is the study design appropriate and does the work have academic merit?

Partly

Is the work clearly and accurately presented and does it cite the current literature?

Partly

If applicable, is the statistical analysis and its interpretation appropriate?

No

Are all the source data underlying the results available to ensure full reproducibility?

Yes

Are the conclusions drawn adequately supported by the results?

Partly

Are sufficient details of methods and analysis provided to allow replication by others?

Yes

Reviewer Expertise:

Offshore wind optimization

I confirm that I have read this submission and believe that I have an appropriate level of expertise to confirm that it is of an acceptable scientific standard, however I have significant reservations, as outlined above.

## References

[1] : Optimal Design for Offshore Wind Farm considering Inner Grid Layout and Offshore Substation Location. *IEEE Transactions on Power Systems* .2017;32(3) : 10.1109/TPWRS.2016.2593501 2041-2048 10.1109/TPWRS.2016.2593501

[2] : A Two-Layer Hybrid Optimization Approach for Large-Scale Offshore Wind Farm Collector System Planning. *IEEE Transactions on Industrial Informatics* .2021;17(11) : 10.1109/TII.2021.3056428 7433-7444 10.1109/TII.2021.3056428

[3] : Optimization and Reliability Evaluation of an Offshore Wind Farm Architecture. *IEEE Transactions on Sustainable Energy* .2017;8(2) : 10.1109/TSTE.2016.2609283 542-550 10.1109/TSTE.2016.2609283

[4] : Collector System Topology for Large-Scale Offshore Wind Farms Considering Cross-Substation Incorporation. *IEEE Transactions on Sustainable Energy* .2020;11(3) : 10.1109/TSTE.2019.2932409 1601-1611 10.1109/TSTE.2019.2932409

# Response

**Published:** 2024-04-16

**Authors:** Ramon Abritta Aguiar Santos

Dear Dr. Xinwei Shen, Since it has been around 1.5 months since you submitted your review report, we (the authors) would like to add this comment to avoid giving the impression that we left your comments unattended. We have been putting significant effort into improving the paper and addressing your comments. Thanks to your suggestions, we identified a flaw in the method, which had not been detected in the initial submission since the effects of the flaw were negligible in the small wind farm under analysis. Open Research Europe has not yet given us the "green light" to submit a new version. Apparently, we need to wait for at least one additional report from another reviewer. We are eager to present the next version of the paper, and we hope this happens soon. We appreciate your valuable feedback. Thank you. Regards,

 Ramon

# Response

**Published:** 2024-04-25

**Authors:** Ramon Abritta Aguiar Santos

Dear Dr. Xinwei Shen, Thanks to your suggestions, we identified a flaw in the proposed methodology. The flaw went unnoticed in the first version of the paper since its consequences are negligible in small wind power plants. This new version addresses larger plants, which enabled us to identify the mistake and amend the method. We have put significant effort into addressing your suggestions, which we believe have helped us develop a substantially better paper. Ahead are some comments about each item you have reported in your review: (1) the literature review has been extended. In the new version, references 13, 14, 15, 19, and 21 are new, which include your recommendations; (2) the problem formulation has been entirely replaced by one that has been reported to be faster in references 13 and 18. The new explanations in Section 2.1, in combination with Figure 1, seek to describe how the cable choices are made; (3) in the new mathematical formulation, Equation 6 addresses power flow conservation; (4) the proposed PCR intends to address reliability while reducing mathematical complexity in the optimization process compared with stochastic approaches. In Section 1, immediately below the bullet points for contributions, you can find a new paragraph explaining why we have chosen the PCR approach combined with Monte Carlo-based assessments; (5) this new version addresses four wind power plants, including London Array, which has 175 wind turbines and 2 substations. The obtained results demonstrate the scalability of the proposed framework. Thank you for your valuable recommendations. Regards, the authors.
